# Classifying American Society of Anesthesiologists Physical Status With a Low-Rank–Adapted Large Language Model: Development and Validation Study

**DOI:** 10.2196/89540

**Published:** 2026-04-21

**Authors:** Min-Chia Chen, Shanq-Jang Ruan, Jo-Hsin Wu, Pei-fu Chen

**Affiliations:** 1 Department of Electronic and Computer Engineering National Taiwan University of Science and Technology Taipei Taiwan; 2 Department of Anesthesiology Far Eastern Memorial Hospital New Taipei Taiwan; 3 Department of Anesthesia University of Iowa Iowa City, IA United States

**Keywords:** American Society of Anesthesiologists Physical Status, large language models, low-rank adaptation, parameter-efficient fine-tuning, electronic health records, natural language processing.

## Abstract

**Background:**

The American Society of Anesthesiologists Physical Status (ASA-PS) classification is integral to preoperative risk assessment; yet, assignment remains subjective and labor-intensive. Recent large language models (LLMs) process free-text electronic health records (EHRs), but few studies have evaluated parameter-efficient adaptations that both predict ASA-PS and provide clinician-readable rationales. Low-rank adaptation (LoRA) is a parameter-efficient technique that updates only a small set of add-on parameters rather than the entire model, enabling efficient fine-tuning on modest data and hardware. A lightweight, instruction-tuned LLM with these capabilities could streamline workflow and broaden access to explainable decision support.

**Objective:**

This study aimed to develop and evaluate a LoRA–fine-tuned large language model meta-AI (LLaMA-3) for ASA-PS classification from preoperative clinical narratives and benchmark it against traditional machine learning classifiers and domain-specific LLMs.

**Methods:**

Preoperative anesthesia notes and discharge summaries were extracted from the EHR and reformatted into an Alpaca-style instruction-response prompt, requesting ASA-PS class labels (I-V) annotated by anesthesiologists. The LoRA-enhanced LLaMA-3 model was fine-tuned with mixed-precision training and evaluated on a hold-out test set. Baselines included random forest classifier, Extreme Gradient Boosting (XGBoost) classifier, support vector machine, fastText, BioBERT, ClinicalBERT, and untuned LLaMA-3. Performance was assessed with micro- and macroaveraged *F*_1_-score and Matthews correlation coefficient (MCC), each reported with 95% bootstrap CIs. Pairwise model error rates were compared using McNemar test.

**Results:**

The LoRA-LLaMA-3 model achieved a micro–*F*_1_-score of 0.780 (95% CI 0.769-0.792) and an MCC of 0.533 (95% CI 0.518-0.546), outperforming other LLM baselines. After fine-tuning, BioBERT reached a micro–*F*_1_-score of 0.762 and an MCC of 0.508, whereas ClinicalBERT achieved a micro–*F*_1_-score of 0.757 and an MCC of 0.515. fastText yielded a micro–*F*_1_-score of 0.762 and an MCC of 0.536. The untuned LLaMA-3 performed poorly (micro–*F*_1_-score of 0.073; MCC of 0.002). However, macro–*F*_1_-score of LoRA-LLaMA-3 (0.316) was lower than that of other language models (0.349-0.372). Among all models, XGBoost obtained the highest scores (micro–*F*_1_-score of 0.815, 95% CI 0.804-0.826; macro–*F*_1_-score of 0.348, 95% CI 0.334-0.361; MCC 0.613, 95% CI 0.599-0.626). Ablation experiments identified dropout = 0.3, learning rate = 3×10^-5^, temperature = 0.1, and top-*P*= 0.1 as the optimal hyperparameter settings. The LoRA model also produced rationales that highlighted medically pertinent terms.

**Conclusions:**

LoRA fine-tuning improved LLaMA-3 from near-random performance into an ASA-PS classifier with higher micro–*F*_1_-score and significantly lower misclassification rates than other language model baselines. However, macroaveraged performance was lower, indicating limited discrimination for minority ASA classes. Traditional machine learning models demonstrated higher predictive performance. Beyond predictive performance, LoRA-LLaMA-3 generated clinician-oriented explanations that enhance decision transparency. By reformatting routine EHR narratives into instruction-response pairs and relying on lightweight parameter adaptation, this approach offers a practical, resource-efficient framework for introducing explainable LLMs to clinical classification tasks.

## Introduction

The American Society of Anesthesiologists Physical Status (ASA-PS) classification system is an essential tool for preoperative assessment, providing a standardized framework to evaluate a patient’s health status and predict perioperative risks. This system consists of 6 levels, ranging from ASA I (a healthy patient) to ASA VI (a brain-dead patient), with an additional “E” designation to indicate emergency surgeries. While the ASA-PS system is widely adopted in clinical practice, it has certain limitations, the most notable being its reliance on subjective judgment. This reliance can lead to inconsistencies among evaluators and variability in risk stratification outcomes. These inconsistencies highlight the need for more objective and reproducible methods for assigning ASA-PS classifications [1,2].

Recent advances in machine learning (ML) and natural language processing (NLP) offer potential solutions to address this challenge. With the widespread adoption of electronic health records (EHRs), large amounts of unstructured clinical text data, including preoperative evaluation notes written by clinicians and anesthesiologists, are now available. These notes contain detailed narratives of patients’ medical and surgical histories, offering insights into their health and perioperative risks [3,4]. Unlike traditional keyword-based approaches, modern NLP techniques, particularly large language models (LLMs), capture contextual nuances and interword dependencies across entire text sequences, demonstrating state-of-the-art performance in text classification tasks [5,6].

Various studies have explored the application of ASA-PS classifications to assess their importance in perioperative risk prediction. Some studies have focused on developing tools to improve interrater consistency, such as introducing more detailed evaluation guidelines [1,2]. However, these approaches remain limited by the subjective nature of the evaluations. Recent research has attempted to automate classification by leveraging structured data in electronic medical records. Nonetheless, these methods often require predefined features and fail to fully use the latent information within unstructured text [3]. In parallel, large-scale EHR studies have derived machine learning ASA scores to support preoperative screening and risk stratification, showing strong discrimination for outcomes such as 30-day mortality and intensive care unit admission [7]. Earlier structured data models have also demonstrated that ASA-PS can be predicted at scale from routinely collected variables, underscoring its central role in perioperative risk models [8].

In the field of NLP, ML models have been widely applied to medical text processing. Early studies used traditional methods such as random forests and support vector machines (SVMs) with n-grams for text classification. However, their performance was constrained by the efficiency of manual feature extraction [9,10]. Subsequent research introduced embedding-based techniques such as fastText, which significantly improved classification accuracy by capturing semantic relationships between words [11,12]. Building on this, NLP systems that read preoperative narratives have directly predicted ASA-PS from free text, demonstrating the feasibility and accuracy of text-only approaches in real clinical documents [13,14]. More recently, transformer-based models have achieved performance comparable with board-certified anesthesiologists for ASA-PS assignment, suggesting a path to reduce interrater variability with automated, scalable tools [15].

Recently, deep learning models such as BioBERT and ClinicalBERT, designed explicitly for medical text, have achieved breakthrough performance in various clinical NLP tasks [5,16]. These models leverage pretrained language models on large-scale medical corpora, showcasing excellent text classification capabilities [17,18]. However, such methods often require substantial computational resources and have limited adaptability to small datasets.

Low-rank adaptation (LoRA) is a parameter-efficient fine-tuning technique that enables pretrained models to adapt to specific tasks with minimal computational overhead [19]. It is particularly suitable for clinical applications with common data sparsity and resource constraints [20]. LoRA updates only a small number of trainable parameters while keeping the majority of the pretrained weights frozen. This design greatly reduces GPU memory requirements and training time [19].

To address the limitations of existing approaches, this study investigates the application of LoRA fine-tuned LLM for predicting ASA-PS classifications from unstructured preoperative evaluation notes. This approach aims to improve the accuracy and consistency of ASA-PS classifications while reducing reliance on subjective human evaluation. Furthermore, this study evaluates whether parameter-efficient fine-tuning of small LLMs can provide clinically interpretable and scalable decision support tools for perioperative risk assessment.

## Methods

### Ethical Considerations

This retrospective single-center study using routinely collected preoperative assessments from November 21, 2015, to August 1, 2023, was approved by the institutional review board of Far Eastern Memorial Hospital (114213-E), a tertiary academic medical center, with a waiver of informed consent granted because the research involved secondary analysis of existing records, posed no more than minimal risk, and could not be practicably conducted without the waiver. The dataset was derived from the institutional EHR system, including routinely documented preoperative anesthesia assessment records for surgical patients. Data were deidentified prior to analysis and analyzed on secure on-premises infrastructure with restricted, audited access. No participant compensation was provided. The manuscript and supplements contain no identifiable images.

### Inclusion and Exclusion Criteria

Cases included in this study were the patients aged 18 years or older and had undergone at least 1 surgical procedure under general or neuraxial anesthesia (N=118,274). Cases classified as ASA VI (brain-dead patients) were excluded (n=90) to ensure relevance to general clinical surgical risk assessment. For patients with multiple visits for each surgery, only the most recent version of the preoperative assessment record was retained. The most recent assessment was selected because it typically reflects the most complete and updated comorbidity documentation. Continuous physiologic and laboratory variables were extracted exclusively from this most recent assessment and were not longitudinally aggregated across prior encounters.

**Figure 1:**
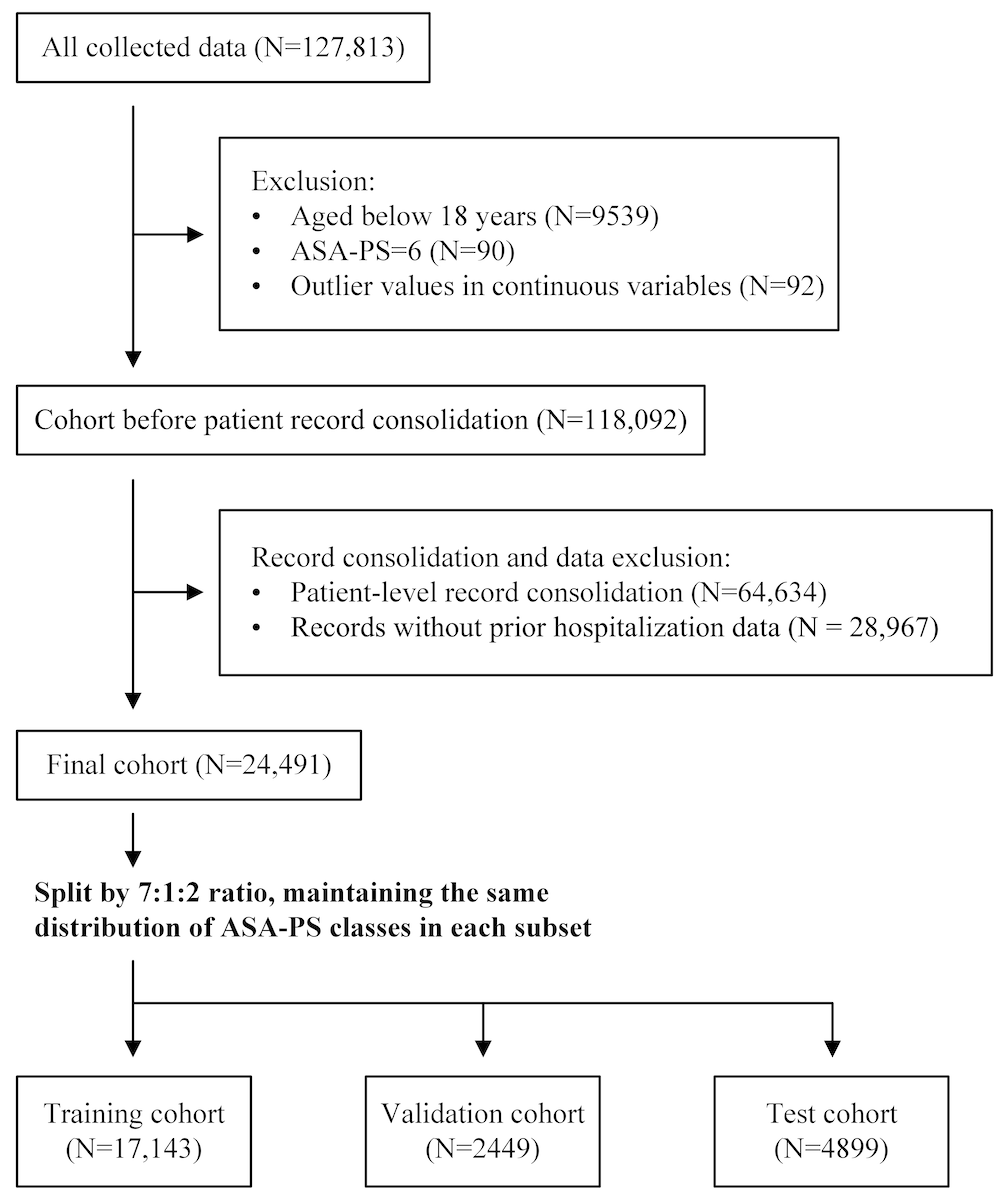
Flow diagram. Outliers were defined as weight less than 25 kg or more than 200 kg (n=21), height less than 120 cm or more than 250 cm (n=70), and hematocrit greater than 75 % (n=1). These values were considered data entry errors and excluded from the analysis. ASA-PS: American Society of Anesthesiologists Physical Status.

The final cohort consisted of 24,491 patients and was randomly split into training (17,143 cases, 70.0%), validation (2449 cases, 10.0%), and test (4899 cases, 20.0%) cohorts using stratification on ASA-PS classes to preserve class distributions across splits (Figure 1). Eligible cases required complete preoperative assessment documentation including clinical narrative and structured perioperative variables used for model input. All eligible cases during the study period were included to maximize model training robustness. Because this study focused on model development rather than hypothesis testing, formal prospective power calculation was not performed. Cases with physiologically implausible values were considered data entry errors and excluded (n=92; Figure 1).

### Outcome Definition

The ASA-PS Classification System is a widely adopted framework for assessing patients’ preoperative physical condition and stratifying perioperative risk [1]. It provides a standardized means of communicating a patient’s overall health status among clinicians, serving as a useful predictor of surgical outcomes and anesthesia-related complications. The classification spans 6 levels, reflecting a progressive increase in systemic disease severity and physiological compromise. Despite its simplicity, the ASA-PS system has demonstrated significant clinical value in risk assessment, particularly when combined with other perioperative variables. Definitions and representative examples for each ASA-PS class are summarized in Table S1 in Multimedia Appendix 1.

### Data Preparation

This study used features extracted from preoperative anesthesia assessment records, including patient demographics, comorbidities, vital signs, laboratory results, surgical and anesthesia records, and discharge notes. Demographic and physiological variables included age, sex, BMI, consciousness status, heart rate, blood pressure, oxygen saturation, body temperature, and respiratory rate. Comorbidities included diabetes, hypertension, hyperlipidemia, cerebrovascular disease, cardiac and pulmonary conditions, liver and kidney disorders, bleeding tendency, smoking history, and prior major surgeries. Laboratory tests included hemoglobin, platelet count, coagulation markers, liver and renal function tests, blood glucose, and serum electrolytes. Text reports of chest radiography, electrocardiogram, and other special examinations (eg, echocardiography and spirometry) were also included.

Surgical and anesthetic features included the surgical department, type of surgery, anesthesia plan, preoperative diagnosis, and anesthetic route. Information on any special conditions noted by anesthesiologists and records of previous intraoperative adverse events were also included. Additionally, free-text fields from previous discharge notes, such as the chief complaint, present illness, admission diagnosis, diagnostic summary, and treatment plan, were extracted and preprocessed by removing line breaks, nonalphanumeric characters, and standard English stopwords using the Natural Language Toolkit [21]. These cleaned narratives were used as unstructured input for model training. An overview of all included features is provided in Table 1. Clinical narratives were primarily written in English, with occasional Traditional Chinese text appearing within the same note (0.4% of total characters across all input documents; median 0.1% per document).

**Table 1:** Feature groups included in the models^a^.

Feature type			Feature classes
**Patient characteristics**			
	Continuous	Age, BMI, blood pressure, heart rate, body temperature, respiratory rate, and oxygen saturation	
	Categorical	Sex (n=2) and consciousness status (n=2)	
**Comorbid conditions**			
	Categorical	Diabetes mellitus (n=2), hyperlipidemia (n=2), hypertension (n=2), cerebrovascular accident (n=2), cardiac disease (n=2), chronic obstructive pulmonary disease (n=2), asthma (n=2), hepatic disease (n=2), renal disease (n=2), bleeding disorder (n=2), history of major operations (n=2), smoking history (n=2), and drug allergy (n=2)	
**Preoperative laboratory values**			
	Continuous	Hemoglobin, platelet count, international normalized ratio, creatinine, aspartate transaminase, alanine transaminase, blood sugar, serum sodium, and serum potassium	
**Preoperative examination reports**			
	Free text	Chest radiography, electrocardiogram, and other special examinations	
**Surgical characteristics**			
	Categorical	Surgical department (n=22), anesthesia type (n=4), emergency level (n=4), and surgery category (n=4)	
	Free text	Preoperative diagnosis, proposed procedure, anesthesia plan, special conditions, and prior adverse anesthesia events	
**Previous discharge notes**			
	Free text	Chief complaint, present illness, admission diagnosis, diagnosis summary, treatment plan, and record time stamp	

^a^Numbers within parentheses indicate the category numbers.

**Figure 2:**
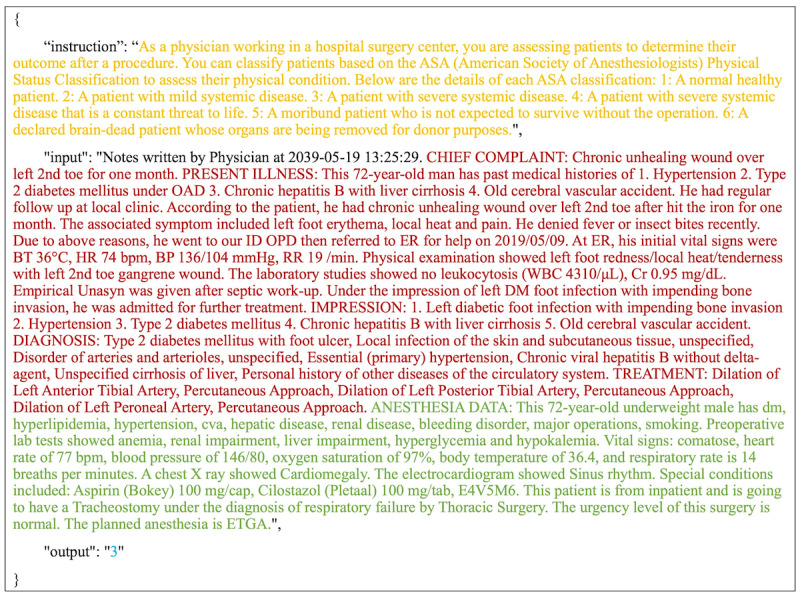
Example of an Alpaca-style prompt used for American Society of Anesthesiologists Physical Status (ASA-PS) classification. The prompt is color-coded to illustrate the data structure and model input components: yellow indicates the role assignment and task instruction (eg, physician's role and the purpose of classification); red represents unstructured clinical narratives (eg, chief complaint, present illness, diagnosis, and treatment); and green highlights structured data elements extracted from electronic health records (eg, laboratory values, vital signs, and comorbidities). In this dataset, the structured field labeled “Special conditions” may include current medications or perioperative notes derived from the original preoperative assessment documentation and was preserved during narrative reconstruction to maintain clinical context. Blue denotes the expected model output format. ASA VI was listed for completeness but not used in training or evaluation.

To adapt this dataset for LLMs, we transformed structured and unstructured fields into narrative-style inputs. Relevant features—such as the chief complaint, present illness, diagnosis, treatment plan, anesthesia notes, and preoperative findings—were reformulated into declarative sentences and concatenated into a single clinical paragraph. This format simulates how anesthesiologists typically synthesize patient records in natural language, as illustrated by the red and green text in Figure 2.

For structured variables (eg, laboratory values, vital signs, and BMI), raw numeric measurements were converted into clinically interpretable text descriptors using predefined reference ranges and heuristic rules (eg, “anemia” and “renal impairment”). These rules were applied prior to model input and are detailed in Table S2 in Multimedia Appendix 1. Accordingly, the language model operated on pregenerated diagnostic descriptors rather than directly interpreting raw numeric physiologic values. When laboratory values were unavailable, the corresponding descriptors were recorded as “normal,” constituting rule-based imputation. Emergency status modifiers (ASA “E”) were not included as input features or classification targets in this study.

For model training and inference, we adopted the Alpaca-style [22] instruction-tuning format. Each sample consisted of three fields: (1) an instruction describing the classification task (ie, predicting ASA-PS), (2) an input with the patient summary, and (3) an output with the ground truth ASA-PS class (I-V) assigned by anesthesiologists. This format helps direct the model’s focus on the classification goal and facilitates consistent training behavior. An example of the training prompt format is shown in Figure 2.

This format provides a standardized, instruction-following setup for LLMs and enables flexible integration of both structured and unstructured data in a clinically realistic manner.

### Model Architecture and Fine-Tuning

To apply LLMs for the classification task of ASA-PS grading, we used the parameter-efficient LoRA [19], which retains general language competence while adapting efficiently to task-specific patterns in limited clinical datasets. We selected Meta-Llama-3-8B-Instruct [23], an instruction-tuned, decoder-only Transformer with approximately 8 billion parameters, as the backbone model and further adapted to ASA-PS classification via LoRA. The base model has already been instruction-tuned on a large, diverse corpus to follow natural language prompts, giving it strong comprehension of complex clinical narratives. These properties make it well suited for analyzing perioperative notes that blend structured elements (vital signs and laboratory values) with free-text observations.

Model training and inference were conducted on the Taiwan Web Service Center platform, developed and operated by the National Center for High-performance Computing. All data preprocessing (see “Data Preparation” section) and model training were performed within this secure environment, ensuring that no data were transferred to external commercial servers. This setup enhances data privacy, computational efficiency, and administrative control, offering advantages over commercial application programming interface–based solutions.

For fine-tuning, we applied the LoRA technique by inserting trainable low-rank matrices into the sublayers of the transformer model, specifically in the attention and feed-forward layers [19].The inserted matrices *W_A_* ∈ *R^r^*^×^*^d^* and *W_B_* ∈ *R^d^*^×^*^r^* form the trainable update term Δ*W*=*W_B_ W_A_*, which is added to the original pretrained weight matrix *W* ∈ *R^d^*^×^*^d^*. Only the LoRA parameters are updated during training, while the original model parameters remain frozen. This design enables efficient adaptation while reducing memory usage and computational cost, making it particularly suitable for clinical applications with limited resources.

**Figure 3:**
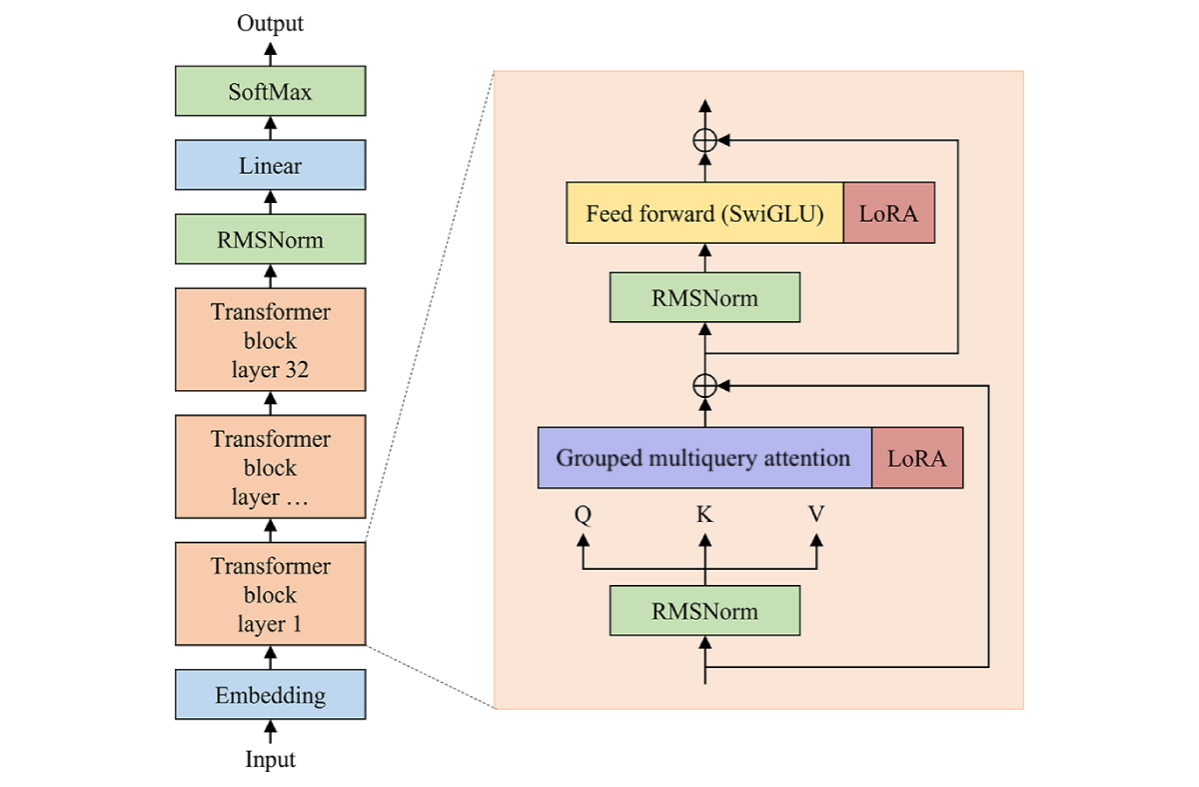
Low-rank adaptation module integration in the LLaMA transformer architecture. Low-rank adaptation modules are inserted into the query and value projections of the grouped multiquery attention and the SwiGLU feed-forward layers (a “switch” gated linear unit that mixes 2 linear projections through a gating mechanism) of each transformer block, operating in parallel with the residual connections. RMSNorm, placed before both attention and feed-forward layers, normalize inputs by their root-mean-square values instead of the full mean and variance, lowering computational cost while preserving stability. LoRA: low-rank adaptation.

As shown in Figure 3, the LoRA modules are integrated into each transformer block of the large language model meta-AI (LLaMA) model, specifically within the grouped multi-query attention (Q/V projections) and SwiGLU feed-forward layers (a “switch” gated linear unit that mixes two 2 linear projections through a gating mechanism) [19]. These modules operate in parallel with the residual connections, ensuring the model’s modularity, scalability, and transferability.

The model input consisted of clinical narratives processed according to the procedure described in the “Data Preparation” section. Inputs were formatted following the Alpaca instruction template, containing 3 fields: instruction, input, and output (Figure 2). The tokenized text was fed into the LoRA-enhanced LLaMA [23] model to predict 1 of the 5 ASA-PS classes.

For model training, we used the Hugging Face accelerate framework [24] and distributed the training across 4 NVIDIA A100 GPUs on the Taiwan Web Service Corporation platform. Mixed-precision (fp16) training [25] was used to optimize memory usage. The batch size was set to 1, with a gradient accumulation step size of 8. The learning rate was set to 3×10^–5^, and a cosine learning rate scheduler along with a LoRA dropout of 0.3 was used for regularization. Training was performed for 1 epoch, with evaluation and checkpointing every 100 steps. The final model selected was the one achieving the best performance on the validation set.

The reason why we had only 1 epoch is due to the strong prior knowledge embedded in the base model, Meta-LLaMA-3-8B-Instruct [23]. As a large-scale instruction-tuned language model, it already possesses extensive general-domain and instruction-following capabilities. Combined with the relatively limited size of our domain-specific dataset, we observed that the model’s performance on the validation set converged quickly and remained stable after a single epoch. Consequently, additional epochs did not yield noticeable improvements and would have increased computational cost unnecessarily.

### Model Evaluation and Statistical Analysis

The LoRA-fine-tuned LLaMA-3 (LLaMA-3-LoRA) and naïve LLaMA-3 were evaluated against several baseline models, including traditional classifiers (Random Forest [10], SVM [9], and Extreme Gradient Boosting [XGBoost] [26]), lightweight embedding-based models (fastText [11]), and biomedical pretrained language models (BioBERT [16] and ClinicalBERT [5]). For comparability, all models consumed the same concatenated instruction + input text; Bidirectional Encoder Representations from Transformers (BERT) models were truncated to 512 tokens and LLaMA-3 formatted in Alpaca style (Figure 2; Multimedia Appendix 2).

For BioBERT and ClinicalBERT, both pretrained and fine-tuned versions were evaluated. Fine-tuning was conducted using Hugging Face’s Trainer with a learning rate of 2×10^–5^, batch size of 8, weight decay of 0.01, and 10 training epochs. The model selection was based on the best microaveraged *F*_1_-score on the validation set. Tokenization was performed using the AutoTokenizer with a maximum input length of 512. When the concatenated instruction and clinical input text exceeded this limit, sequences were truncated by retaining the first 512 tokens, consistent with standard BERT input handling. No sliding-window or hierarchical chunking strategies were applied. The final classification head was trained using cross-entropy loss on 5 ASA-PS classes. Fine-tuned models are denoted by appending the suffix -ft to the original model name (eg, BioBERT-ft and ClinicalBERT-ft).

The traditional ML baselines (Random Forest, SVM, and XGBoost) were trained using the term frequency inverse document frequency features derived from the concatenation of instruction and input fields. To optimize performance, we applied random search with 5-fold cross-validation to identify the best hyperparameter configurations. For Random Forest, the final model used 300 estimators, a maximum depth of 20, a maximum feature setting of “sqrt,” and a minimum samples split of 5. The best-performing SVM model used a radial basis function kernel with C set to 10 and gamma set to 0.01. The final XGBoost configuration used a maximum depth of 6, a learning rate of 0.1, 100 estimators, and subsample and “colsample_bytree” (the subsample ratio of columns when constructing each tree) values of 0.8. Hyperparameter selection was based on the best cross-validated *F*_1_-score on the training set.

Models were evaluated using micro- and macroaveraged area under the receiver operating characteristic curve (AUROC), area under the precision-recall curve (AUPRC), accuracy, *F*_1_-score, and the Matthews correlation coefficient (MCC). MCC incorporates all cells of the confusion matrix and therefore remains stable under class imbalance [27]. Given the class imbalance in multiclass ASA-PS, *F*_1_-score and MCC were emphasized when comparing models [27,28]. All performance metrics were calculated based on exact agreement between predicted ASA-PS class and the clinician-assigned reference label. Because ASA-PS represents an ordinal clinical scale, adjacent and distant misclassification patterns were additionally analyzed and reported separately to provide clinically interpretable error characterization.

Performance metrics were reported with 95% CIs derived from 1000 bootstrap resamples of the test set. For pairwise comparisons between classifiers evaluated on the same test set, we used McNemar test, which is appropriate for paired comparisons on identical samples. For each model pair, predictions were collapsed into binary outcomes (correct vs incorrect relative to the reference ASA-PS label), and a 2×2 contingency table was constructed. Statistical significance was assessed using McNemar test with continuity correction. Because McNemar test evaluates differences in classification error rates, it was used to assess overall prediction disagreement between models rather than metric-specific differences. To control the family-wise error rate across multiple pairwise comparisons, we applied a Bonferroni adjustment, setting the significance threshold at .05/*m*, where .05 is the α level and *m* is the number of model pairs. All models were evaluated on the same fixed test split to ensure comparability. Baseline characteristics across the training, validation, and test cohorts were compared using the Kruskal-Wallis test for continuous variables and the chi-square test for categorical variables. These tests were performed as distributional checks because cohorts were generated by stratified random splitting.

### Context-Length Controlled Evaluation

To ensure that model performance was not confounded by differences in input context windows, we conducted a control experiment using only test cases whose concatenated instruction and clinical input text contained 512 tokens when tokenized using the standard BERT tokenizer (n=694). This subset ensured identical information availability across architectures without requiring truncation. All models were reevaluated using the same text inputs to isolate the effect of model architecture and fine-tuning strategy from input length differences.

### Ablation Analysis of Hyperparameter Effects in LLaMA-3-LoRA

Ablation experiments were conducted to systematically assess how key hyperparameters affect model performance. Each experiment varied a single parameter while keeping the others fixed to isolate its individual contribution. The parameters evaluated included (1) dropout rates (probability of randomly zeroing hidden units to mitigate overfitting): 0.1, 0.2, 0.3, 0.4, and 0.5; (2) learning rates (gradient-descent step size): 1×10^–5^, 3×10^–5^, 5×10^–5^, and 8×10^–5^; (3) temperature (logit-scaling factor that controls output randomness): 0.1, 0.3, 0.5, 0.7, and 0.9, with *top_p* fixed at 1.0; and (4) *top_p* (nucleus-sampling threshold that limits token selection to the smallest set whose cumulative probability ≤ *P*): 0.1, 0.3, 0.5, 0.7, and 0.9, with temperature fixed at 1.0. This design minimized interaction effects between sampling parameters and ensured that observed changes could be attributed to the parameter of interest.

All experiments used the same data preprocessing pipeline (see “Data Preparation” section), model initialization, and LoRA fine-tuning configuration (see “Model Architecture and Fine-Tuning” section) as in the main experiments. This setup ensured that results were directly comparable and that performance shifts could be confidently linked to the hyperparameter changes being tested.

### Attention-Based Analysis of Model Focus

We analyzed the attention weights from the final transformer layer of the LoRA-fine-tuned LLaMA-3 model in order to better understand the internal behavior during classification [23]. The self-attention scores were aggregated across all heads and averaged to represent interaction strengths between token groups [29]. Subword tokens were merged into phrases using white space as a delimiter, and the top 30 phrase pairs with the highest attention were selected. These relationships were visualized using a lower triangular heatmap to reveal attention clusters among clinically meaningful terms. Additionally, attention values directed toward the final output token were collected to rank input phrases based on their contribution to the predicted ASA-PS class.

### Model-Generated Explanation

The model was also configured to generate natural language rationales for its ASA-PS classification output. For this purpose, we used an expanded Alpaca-style prompt that appended an explicit request for free-text reasoning to the original classification instruction, while retaining the same clinical narrative input structure (Table S3 in Multimedia Appendix 1). This prompt variant was used exclusively for explainability analysis and not for performance evaluation. The generated explanation commonly referenced key clinical factors such as age, comorbidities, and procedure-related concerns [30]. Representative examples were extracted and qualitatively assessed to verify whether the generated rationales aligned with clinically valid reasoning.

### Blinded Evaluation of Generated Rationales

To assess the reliability of model-generated explanations, we conducted a blinded qualitative evaluation using a random sample of cases from the held out test set. A total of 40 cases were randomly selected and stratified to cover ASA-PS classes I through IV. For each case, reviewers were provided with the original clinical narrative and the corresponding model-generated rationale, while predicted ASA class and model identity were blinded. Model outputs were presented in randomized order and deidentified format across cases to minimize potential recognition of model identity.

Rationales were independently evaluated by 2 board-certified anesthesiologists using a predefined assessment form. Reviewers rated (1) factual accuracy using a 3-level scale (no hallucination, minor hallucination [unsupported detail not affecting ASA reasoning], or major hallucination [clinically incorrect or potentially misleading for ASA classification]), and (2) clinical coherence with ASA-PS classification principles (yes or no). Rationale quality was summarized descriptively using proportions across predefined evaluation categories.

Summary statistics were calculated as pooled proportions across raters. Interrater agreement was assessed using weighted k for factual accuracy and unweighted Cohen κ for clinical coherence. Because k statistics can be influenced by prevalence and category distribution, Gwet’s first-order agreement coefficient (AC1) was additionally calculated as a complementary agreement measure [31].

### Software and Computational Environment

All models were implemented in Python (version 3.10.14; Python Software Foundation) and executed on NVIDIA A100 (80 GB) GPUs with CUDA (version 12.1; NVIDIA Corporation) and cuDNN (version 8.9.2; NVIDIA Corporation). LLM fine-tuning used PyTorch (version 2.3.0; PyTorch Foundation), Transformers (version 4.47.1; Hugging Face), Accelerate (version 1.2.0; Hugging Face), PEFT (version 0.11.1; Hugging Face), bitsandbytes (version 0.42.0; bitsandbytes Foundation), and TRL (version 0.8.6; Hugging Face). Baseline models comprised Random Forest and SVM implemented in scikit-learn (version 1.6.1; scikit-learn developers), XGBoost (version 3.0.1; XGBoost developers), fastText (version 0.9.3; fastText project), and BioBERT/ClinicalBERT via Transformers (version 4.47.1; Hugging Face). Statistical analysis and visualization used NumPy (version 2.2.6; NumPy community), pandas (version 2.2.3; Pandas Development Team), Matplotlib (version 3.10.3; Matplotlib Development Team), and Seaborn (version 0.13.2; Seaborn Development Team).

## Results

### Cohort Characteristics

In the overall cohort (N=24,491), the median age of the patients was 60 years (IQR 47-69), with 13,775 (56.25%) cases being male. Regarding ASA-PS classification, most cases were categorized as ASA II (15,272, 62.36% cases) and ASA III (8024, 32.76% cases), followed by ASA I (535, 2.18% cases), ASA IV (606, 2.47% cases), and ASA V (54, 0.22% cases). Additionally, 1200 (4.90%) cases of surgeries were marked as ASA emergency status.

In terms of anesthesia type, 12,648 (51.74%) cases underwent general anesthesia with endotracheal tube, followed by 5290 (21.64%) cases with total intravenous anesthesia and 4038 (16.52%) cases with general anesthesia with mask ventilation. Regarding surgical urgency, most cases underwent elective surgery (21,217, 86.64% cases), while 2531 (10.34%) cases had urgent surgeries, and a smaller percentage required emergency surgery (686 cases, 2.80%) or immediate surgery (55, 0.22% cases).

Furthermore, the most common comorbidities among the study population included hypertension (11,315, 46.20% cases), diabetes (6238, 25.47% cases), and cardiac disease (6279, 25.64% cases). In terms of surgical specialties, the most frequently performed surgeries were in urology (6068, 24.78% cases) and orthopedics (3759, 15.35% cases), followed by general surgery (3094, 12.63% cases) and cardiovascular surgery (2904, 11.86% cases; Table S4 in Multimedia Appendix 1).

### Comparative Evaluation of Models

Among the language models evaluated, the LLaMA-3-LoRA achieved the highest micro–*F*_1_-score of 0.780 (95% CI 0.769-0.792) and MCC of 0.533 (95% CI 0.518-0.546), while the original LLaMA-3 achieved a micro–*F*_1_-score of 0.073 (95% CI 0.066-0.081) and MCC of 0.002 (95% CI 0.001-0.002; Table 2).

**Table 2:** Overall performance comparison across models using microaveraged metricsa.

Model	*F*_1_-score (95% CI)	MCC^b^ (95% CI)	Accuracy (95% CI)	AUROC^c^ (95% CI)	AUPRC^d^ (95% CI)
LLaMA-3-LoRA	0.780 (0.769-0.792)	0.533 (0.518-0.546)	0.780 (0.769-0.792)	0.863 (0.853-0.872)	0.653 (0.639-0.666)
LLaMA-3	0.073 (0.066-0.081)	0.002 (0.001-0.002)	0.073 (0.066-0.081)	0.421 (0.407-0.435)	0.191 (0.180-0.202)
BioBERT-ft	0.762 (0.750-0.774)	0.508 (0.494-0.522)	0.762 (0.750-0.774)	0.852 (0.841-0.861)	0.629 (0.615-0.642)
BioBERT	0.067 (0.060-0.074)	0.062 (0.056-0.062)	0.067 (0.060-0.074)	0.417 (0.403-0.431)	0.191 (0.180-0.202)
ClinicalBERT-ft	0.757 (0.745-0.769)	0.515 (0.501-0.529)	0.757 (0.745-0.769)	0.848 (0.838-0.858)	0.622 (0.608-0.635)
ClinicalBERT	0.624 (0.610-0.637)	0.000 (0.000-0.000)	0.624 (0.610-0.637)	0.765 (0.753-0.777)	0.464 (0.450-0.478)
fastText	0.762 (0.750-0.774)	0.536 (0.522-0.550)	0.762 (0.750-0.774)	0.851 (0.841-0.861)	0.628 (0.614-0.641)
Random Forest	0.794 (0.782-0.805)	0.563 (0.549-0.577)	0.794 (0.782-0.805)	0.871 (0.862-0.880)	0.671 (0.658-0.684)
XGBoost^e^	0.815 (0.804-0.826)	0.613 (0.599-0.626)	0.815 (0.804-0.826)	0.884 (0.875-0.893)	0.701 (0.688-0.714)
Support vector machine	0.809 (0.798-0.820)	0.597 (0.583-0.611)	0.809 (0.798-0.820)	0.880 (0.871-0.889)	0.692 (0.679-0.705)

^a^Accuracy and MCC are reported along with **micro**averaged AUROC, AUPRC, and *F*_1_-score. Values are shown as point estimates with 95% CI obtained via bootstrapping.

^b^MCC: Matthews correlation coefficient.

^c^AUROC: area under the receiver operating characteristic curve.

^d^AUPRC: area under the precision-recall curve.

^e^XGBoost: Extreme Gradient Boosting.

In comparison, fastText achieved lower performance, with a micro–*F*_1_-score of 0.762 (95% CI 0.750-0.774) and MCC of 0.536 (95% CI 0.522-0.550). After fine-tuning on the ASA-PS dataset, BioBERT-ft achieved a micro–*F*_1_-score of 0.762 (95% CI 0.750-0.774) and MCC of 0.508 (95% CI 0.494-0.522). ClinicalBERT-ft obtained similar performance, with a micro–*F*_1_-score of 0.757 (95% CI 0.744-0.770) and MCC of 0.515 (95% CI 0.501-0.529), while their pretrained counterparts performed poorly (BioBERT: micro–*F*_1_-score 0.067, 95% CI 0.060-0.074, and MCC 0.062, 95% CI 0.056-0.062; ClinicalBERT: micro–*F*_1_-score 0.624, 95% CI 0.610-0.637, and MCC 0.000, 95% CI 0.000-0.000).

Among all evaluated models, XGBoost performed best, with a micro–*F*_1_-score of 0.815 (95% CI 0.804-0.826) and MCC of 0.613 (95% CI 0.599-0.626). SVM ranked second with a micro–*F*_1_-score of 0.809 (95% CI 0.798-0.820) and MCC of 0.597 (95% CI 0.583-0.611). Complete metrics for all models are provided in Table S5 in Multimedia Appendix 1.

Although microaveraged metrics reflect overall classification performance, macroaveraged metrics were additionally examined to evaluate class-specific discrimination. LLaMA-3-LoRA achieved a macro–*F*_1_-score of 0.316 (95% CI 0.303-0.329), which was lower than other language model baselines, including BioBERT-ft (0.372), fastText (0.356), and ClinicalBERT-ft (0.349), and also lower than XGBoost (0.348) and SVM (0.322; Table S5 in Multimedia Appendix 1). These findings indicate reduced discriminative performance for minority ASA classes, consistent with the substantial class imbalance in the dataset.

Pairwise comparisons using McNemar test showed significant differences in overall classification error rates between most model pairs (Multimedia Appendix 3). XGBoost showed significantly lower error rates than LLaMA-3-LoRA (*P*<.001), and LLaMA-3-LoRA showed significantly lower error rates than all other language model baselines. After Bonferroni adjustment for 21 pairwise tests (a_(Bonferroni)_ = 0.0024), these differences remained statistically significant except for comparisons between LLaMA-3-LoRA and BioBERT-ft or fastText. Nonsignificant differences were also observed in comparisons between LlaMA-3 and BioBERT, BioBERT-ft and ClinicalBERT-ft, BioBERT-ft and fastText, ClinicalBERT-ft and fastText, and XGBoost and SVM.

**Figure 4:**
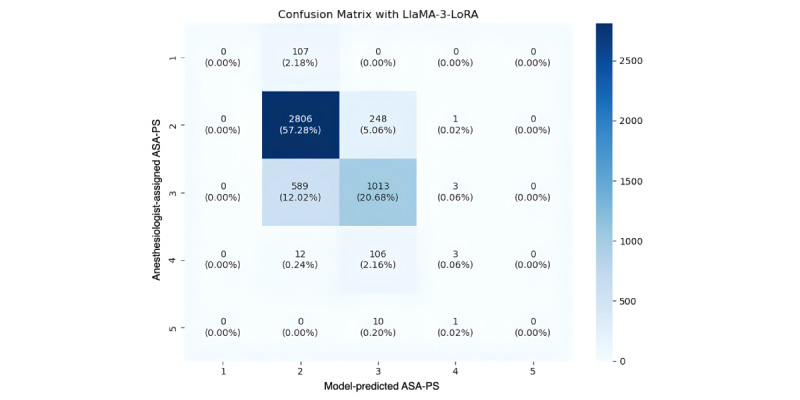
Confusion matrix for ASA-PS classification using the LLaMA-3-LoRA model. Most predictions for the dominant ASA I-III classes were concentrated along the diagonal, indicating primarily adjacent-class misclassifications. Off-diagonal errors were rare except in minority classes (ASA IV-V), reflecting the challenge of extreme class imbalance. ASA-PS: American Society of Anesthesiologists Physical Status.

**Figure 5:**
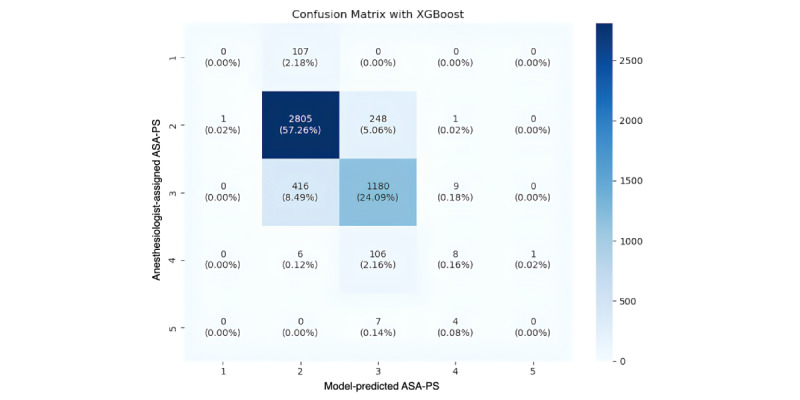
Confusion matrix for ASA-PS classification using the XGBoost model. Most predictions for the major ASA I-III classes were aligned along the diagonal, indicating predominantly adjacent-class misclassifications. Compared with LLaMA-3-LoRA, XGBoost showed slightly fewer adjacent-class errors for ASA II-III, but off-diagonal errors increased for the minority classes (ASA IV-V), reflecting the persistent impact of class imbalance. ASA-PS: American Society of Anesthesiologists Physical Status; XGBoost: Extreme Gradient Boosting.

To further illustrate model error patterns, confusion matrices for LLaMA-3-LoRA and XGBoost were analyzed (Figures 4 and 5). For the clinically most common strata (ASA II and III, and to a lesser extent ASA I), most predictions were concentrated along the diagonal, indicating that misclassifications were primarily between adjacent categories rather than distant ones. Distant errors (2 classes apart) were rare, suggesting that both models captured ordinal relationships among ASA-PS levels. In contrast, minority classes (ie, ASA IV and V) exhibited increased off-diagonal errors across all models, reflecting the expected challenge under severe class imbalance. Compared with LLaMA-3-LoRA, XGBoost showed slightly fewer adjacent-class errors for ASA II-III, but off-diagonal errors increased for the minority classes (ASA IV-V), reflecting the persistent impact of class imbalance.

### Context-Length Controlled Evaluation

Among the evaluated models under the input length ≤512 tokens (n=694), the LLaMA-3-LoRA achieved the highest *F*_1_-score of 0.879 (95% CI 0.855-0.903) and MCC of 0.297 (95% CI 0.263-0.331), SVM achieving an *F*_1_-score of 0.876 (95% CI 0.852-0.901) and MCC of 0.238 (95% CI 0.206-0.269), and XGBoost achieving an *F*_1_-score of 0.873 (95% CI 0.848-0.898) and MCC of 0.198 (95% CI 0.168-0.227). Fine-tuned BERT models followed, with ClinicalBERT-ft and BioBERT-ft obtaining *F*_1_-scores of 0.847 (95% CI 0.820-0.874) and 0.833 (95% CI 0.805-0.861) and MCC values of 0.261 (95% CI 0.228-0.293) and 0.187 (95% CI 0.158-0.216), respectively. fastText showed slightly lower performance, with an *F*_1_-score of 0.814 (95% CI 0.785-0.843) and MCC of 0.202 (95% CI 0.172-0.232). Interestingly, the untuned ClinicalBERT achieved a relatively high *F*_1_-score of 0.869 (95% CI 0.844-0.894) but an MCC of 0.000, suggesting that its predictions were concentrated in a single majority class (ASA-PS class II). Other untuned transformer models, including LLaMA-3 and BioBERT, both performed poorly (*F*_1_-score ≤0.089; Table 3).

**Table 3:** Performance comparison across models under controlled input length (≤512 tokens)^a^.

Model	*F*_1_-score (95% CI)	MCC^b^ (95% CI)	Accuracy (95% CI)	AUROC^c^ (95% CI)	AUPRC^d^ (95% CI)
LLaMA-3-LoRA	0.879 (0.855-0.903)	0.297 (0.263-0.331)	0.879 (0.855-0.903)	0.919 (0.899-0.940)	0.803 (0.773-0.832)
LLaMA-3	0.089 (0.068-0.111)	0.000 (0.000-0.000)	0.089 (0.068-0.111)	0.431 (0.394-0.468)	0.190 (0.161-0.219)
BioBERT-ft	0.833 (0.805-0.861)	0.187 (0.158-0.216)	0.833 (0.805-0.861)	0.889 (0.864-0.911)	0.735 (0.702-0.768)
BioBERT	0.059 (0.042-0.077)	0.000 (0.000-0.000)	0.059 (0.042-0.077)	0.373 (0.336-0.408)	0.239 (0.206-0.269)
ClinicalBERT-ft	0.847 (0.820-0.874)	0.261 (0.228-0.293)	0.847 (0.820-0.874)	0.898 (0.875-0.920)	0.756 (0.723-0.787)
ClinicalBERT	0.869 (0.844-0.894)	0.000 (0.000-0.000)	0.869 (0.844-0.894)	0.913 (0.891-0.933)	0.788 (0.756-0.817)
fastText	0.814 (0.785-0.843)	0.202 (0.172-0.232)	0.814 (0.785-0.843)	0.876 (0.852-0.901)	0.709 (0.675-0.743)
Random Forest	0.870 (0.845-0.895)	0.099 (0.076-0.120)	0.870 (0.845-0.895)	0.914 (0.893-0.934)	0.790 (0.759-0.820)
XGBoost^e^	0.873 (0.848-0.898)	0.198 (0.168-0.227)	0.873 (0.848-0.898)	0.915 (0.894-0.936)	0.794 (0.764-0.824)
Support vector machine	0.876 (0.852-0.901)	0.238 (0.206-0.269)	0.876 (0.852-0.901)	0.917 (0.896-0.937)	0.798 (0.768-0.828)

^a^Accuracy and MCC are reported along with microaveraged AUROC, AUPRC, and *F*_1_-score. Values are shown as point estimates with 95% CI obtained via bootstrapping. See Table S5 in Multimedia Appendix 1 for macroaveraged metrics.

^b^MCC: Matthews correlation coefficient.

^c^AUROC: area under the receiver operating characteristic curve.

^d^AUPRC: area under the precision-recall curve.

^e^XGBoost: Extreme Gradient Boosting.

### Ablation Analysis of Hyperparameter Effects in LLaMA-3-LoRA

#### Dropout

As shown in Table S6 in Multimedia Appendix 1, a dropout rate of 0.4 yielded the best performance (*F*_1_-score 0.782, 95% CI 0.771-0.794; MCC 0.538, 95% CI 0.524-0.552). A slightly lower rate of 0.3 yielded similar results (*F*_1_-score 0.780, 95% CI 0.769-0.792; MCC 0.533, 95% CI 0.518-0.546).

#### Learning Rate

As shown in Table S7 in Multimedia Appendix 1, a learning rate of 3×10^–5^ yielded the best performance (*F*_1_-score of 0.780, 95% CI 0.769-0.792; MCC 0.533, 95% CI 0.518-0.546). In comparison, both a lower rate (1×10^–5^) and a higher rate (8×10^–5^) reduced MCC scores (0.111, 95% CI 0.102-0.119 and 0.440, 95% CI 0.426-0.454, respectively).

#### Temperature

As shown in Table S8 in Multimedia Appendix 1, a temperature of 0.1 yielded the best performance (*F*_1_-score of 0.777, 95% CI 0.765-0.788; MCC 0.526, 95% CI 0.512-0.540). As the temperature increased to 0.9, performance gradually declined, with the MCC dropping to 0.195 (95% CI 0.183-0.206).

#### Top-P (Nucleus Sampling)

As shown in Table S9 in Multimedia Appendix 1, *top_p* values of 0.1 yielded the best performance (*F*_1_-score 0.782, 95% CI 0.771-0.794; MCC 0.538, 95% CI 0.524-0.552), closely followed by 0.3 (*F*_1_-score 0.782, 95% CI 0.770-0.793; MCC 0.536, 95% CI 0.522-0.550). When *top_p* value was raised to 0.5 and 0.9, MCC dropped to 0.161 (95% CI 0.151-0.171) and 0.179 (95% CI 0.168-0.189), respectively.

#### Final Configuration

Subsequent experiments used a dropout rate of 0.3, a learning rate of 3×10^–5^, a temperature of 0.1, and a *top_p* value of 0.1—the combination that maximized MCC and *F*_1_-score during hyperparameter tuning. Although a dropout rate of 0.4 slightly outperformed 0.3, the difference was not statistically significant, and the smaller value (0.3) was retained to mitigate the risk of underfitting and overregularization typically associated with excessive dropout in LLM fine-tuning [32]. This decision is similar with common practice in LoRA fine-tuning (≈0.05-0.1 for LLaMA models) [33].

### Attention-Based Analysis of Model Focus

**Figure 6:**
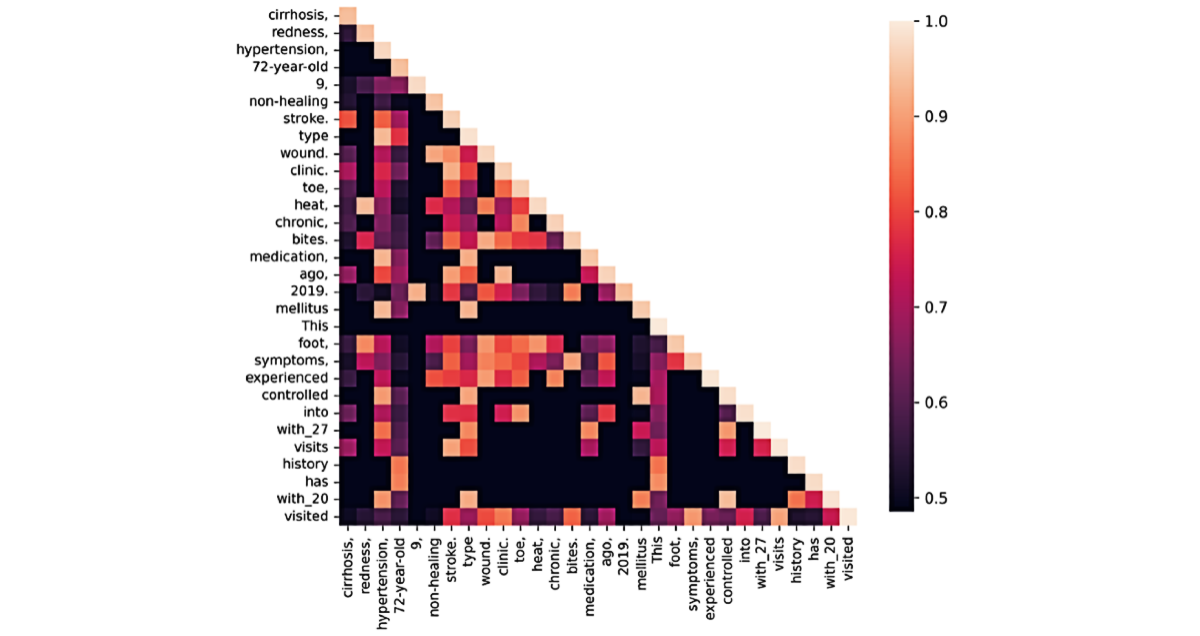
Attention heatmap between top 30-word phrases in the input text for 1 case (ASA-PS class III) from the test cohort. The lower triangular matrix shows average unidirectional attention weights from each phrase to preceding phrases derived from the final transformer layer. Brighter regions indicate stronger attention. Medical terms tend to cluster together, reflecting localized attention focus. Representative attention visualizations for ASA-PS classes II and IV are provided in Multimedia Appendix 4.

**Figure 7:**
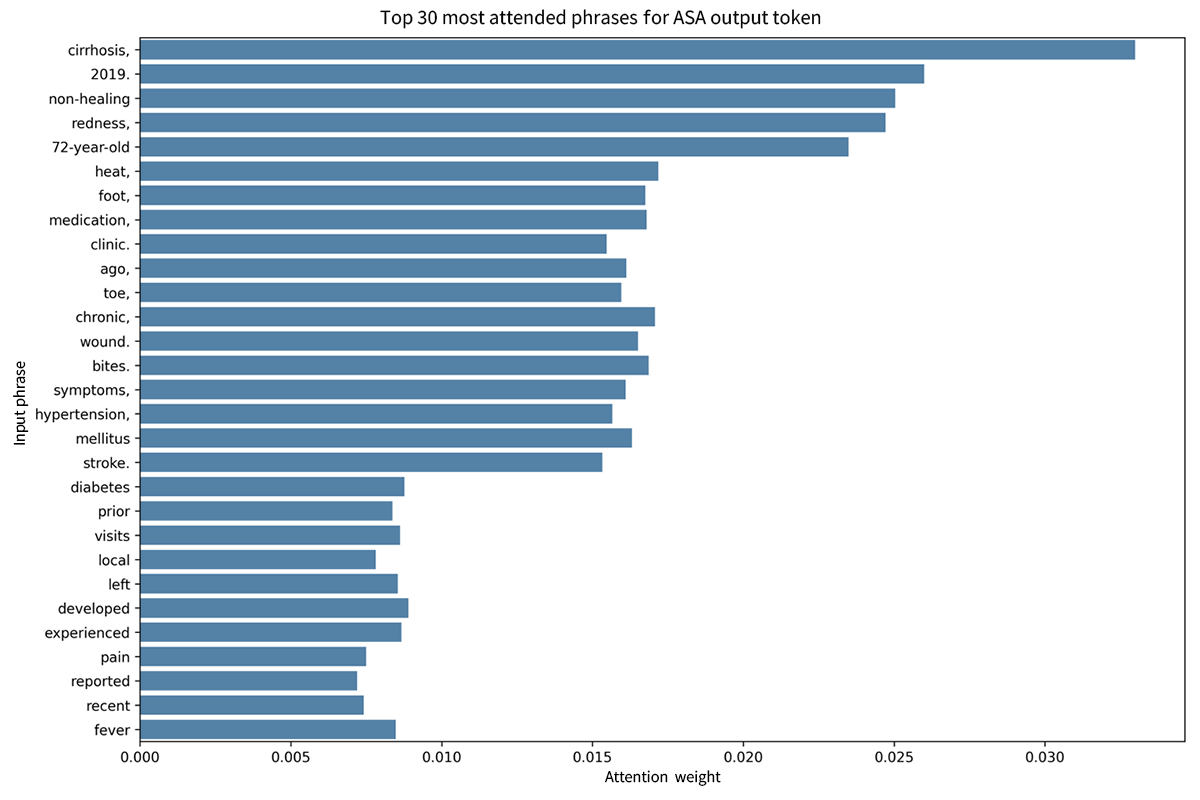
Top 30 most attended phrases contributing to the American Society of Anesthesiologists Physical Status (ASA-PS) output token for 1 case (ASA-PS class III) from the test cohort. Bar height represents cumulative attention directed toward each phrase by the final output token. The highlighted phrases reflect the model’s focus on comorbidities and demographic cues relevant to perioperative risk. Representative attention visualizations for ASA-PS classes II and IV are provided in Multimedia Appendix 4. ASA: American Society of Anesthesiologists.

The attention heatmap for a case of ASA-PS class III (Figure 6) revealed strong intrasection interactions among clinical terms, such as “cirrhosis,” “redness,” and “hypertension,” indicating local contextual coherence. Additionally, Figure 7 visualizes the top 30 input phrases receiving the highest cumulative attention toward the output token for 1 example from the test set, including “cirrhosis,” “nonhealing,” “diabetes,” and “72-year-old,” reflecting the model’s emphasis on comorbidities and age-related indicators during ASA-PS classification.

Comparable attention patterns were observed in additional representative cases from ASA-PS classes II and IV, where attention remained concentrated on comorbidity descriptors (eg, hydronephrosis, cardiomegaly, and encephalopathy) and physiologic abnormalities (eg, hypokalemia and leukocytosis). However, occasional high-attention tokens with limited direct clinical relevance were also observed, reflecting the probabilistic nature of attention mechanisms [34]. Representative visualizations for ASA-PS classes II and IV are provided in Multimedia Appendix 4.

### Model-Generated Explanation

Table S10 in Multimedia Appendix 1 presented 10 model-generated explanations for ASA classes assignment. The illustrative examples were generated using a separate explanation-augmented prompt and do not correspond to the deterministic inference pathway used for quantitative performance evaluation. For class III, the model repeatedly referenced multiple chronic conditions (eg, diabetes, hypertension, coronary artery disease, renal failure, and bleeding disorders), recent cardiac evaluations, and limited mobility due to comorbidities. Explanations for lower classes (I-II) focused on stable or well-managed chronic conditions, preserved organ function, and low surgical risk. In contrast, output for the higher classes (IV-V) highlighted critically ill patients with recent cardiovascular events, hemodynamic instability, or emergency surgical requirements. Full narrative inputs are provided in Table S11 in Multimedia Appendix 1.

### Blinded Evaluation of Generated Rationales

In blinded qualitative evaluation of 40 randomly sampled cases, LLaMA-3-LoRA demonstrated high factual reliability and clinical coherence (Table S12 in Multimedia Appendix 1). Across both raters, LLaMA-3-LoRA rationales contained no hallucination in 86.25% of evaluations, minor hallucination in 7.50%, and major hallucination in 6.25%. Clinical coherence with ASA-PS principles was observed in 91.25% of LLaMA-3-LoRA rationales.

For LLaMA-3-LoRA, Cohen k indicated fair to moderate agreement (κ=0.22-0.44), whereas observed agreement remained high (≥87.5%). Gwet’s AC1 demonstrated substantial to almost perfect reliability (AC1 = 0.85-0.87). The discrepancy between κ and observed agreement likely reflects the high prevalence of top-category ratings, a known limitation of κ statistics [31]. Accordingly, κ and AC1 were reported and interpreted jointly to provide complementary perspectives on interrater agreement (Table S13 in Multimedia Appendix 1).

## Discussion

### Principal Findings

This study aimed to evaluate whether parameter-efficient fine-tuning of a small LLM could provide accurate, reproducible, and clinically interpretable ASA-PS classification from preoperative clinical narratives. From a clinical perspective, our findings suggest that such models may serve as scalable decision support tools to assist ASA documentation and perioperative risk communication.

Our study demonstrates the performance of the LLaMA-3-LoRA model in classifying ASA-PS based on preoperative clinical narratives. The model achieved a microaveraged *F*_1_-score of 0.780 (95% CI 0.769-0.792) and an MCC of 0.533 (95% CI 0.518-0.546), outperforming all baseline language models, including the original, untuned LLaMA-3 (*F*_1_-score 0.073, MCC 0.002), as shown in Table 2. However, macroaveraged performance revealed reduced class-level discrimination. LLaMA-3-LoRA achieved a macro–*F*_1_-score of 0.316 (95% CI 0.303-0.329), which was lower than other language model baselines (0.349-0.372), XGBoost (0.348), and SVM (0.322; Table S5 in Multimedia Appendix 1). This discrepancy likely reflects substantial class imbalance, particularly for minority ASA classes. Pairwise comparisons using McNemar test further showed that XGBoost had significantly lower error rates than LLaMA-3-LoRA (*P*<.001), whereas differences between LLaMA-3-LoRA and other language model baselines were not statistically significant after Bonferroni adjustment (Multimedia Appendix 3).

While traditional ML models such as XGBoost, Random Forest, and SVM achieved higher scores on several discrimination metrics (eg, macro–*F*_1_-score, AUROC, and MCC), they process text through manually engineered token-based features without the semantic understanding offered by contextual embeddings in LLMs. In contrast, our model incorporates unstructured clinical documentation and outputs both predictions and explanations.

The relative advantage of traditional machine learning models in our study may also reflect the structural characteristics of the ASA-PS classification task. Although ASA assignment is documented in narrative clinical notes, much of the discriminative information is conveyed through semistructured clinical descriptors, such as explicit comorbidity labels, physiologic abnormalities, and standardized diagnostic terminology. Such features can be efficiently captured by sparse feature representations and tree-based classifiers. Prior work comparing LLMs with locally trained machine learning models in EHR-based prediction tasks has similarly demonstrated superior performance of traditional ML when predictive signals are dominated by structured or semistructured features [35]. These findings suggest that task structure, rather than model scale alone, plays a critical role in determining comparative model performance.

In addition to aggregate metrics, we examined the classwise misclassification table which separates adjacent (±1 class) from distant (≥2 classes) errors (Table S14 in Multimedia Appendix 1). For the 3 most common categories, ASA-PS classes I, II, and III, the LLaMA-3-LoRA model showed comparable accuracy to the best-performing ML models (eg, XGBoost and SVM): distant errors were essentially absent, and the few mistakes that did occur were almost exclusively adjacent (eg, ASA I cases predicted as ASA II). Such single-step slips are clinically less consequential because the boundaries between neighboring ASA classes are inherently ambiguous. In contrast, the sparsely represented ASA-PS IV and V classes proved challenging for every model, with both adjacent and distant errors rising sharply, an expected consequence of extreme class imbalance. Taken together, these findings suggest that instruction-tuned LLM can approach but did not surpass state-of-the-art machine learning algorithms in class-specific reliability for the clinically prevalent strata, while also offering the added benefits of interpretability and flexibility. Across all evaluated models, prediction of rare ASA classes (I and V) remained poor, with most models concentrating predictions within intermediate severity levels (Multimedia Appendix 5). Similar limitations have been reported in prior ASA classification studies [13,15], suggesting that automated ASA prediction remains intrinsically challenging and may not yet fully operationalizable for extreme severity strata.

In addition, ASA-PS assignment itself is known to demonstrate moderate interrater variability among anesthesiologists [36]. Therefore, some adjacent-class discrepancies between model predictions and recorded ASA labels may reflect clinically acceptable differences in interpretation rather than definitive model error. This inherent uncertainty in the reference standard should be considered when interpreting classification performance, particularly between neighboring ASA categories such as classes II and III.

In a context-length controlled experiment (≤512 tokens), LLaMA-3-LoRA maintained the highest micro–*F*_1_-score of 0.879 (95% CI 0.855-0.903) and MCC of 0.297 (95% CI 0.263-0.331; Table 3). However, under the same input constraint, the smaller domain-specific BioBERT-ft achieved higher macro–*F*_1_-score (0.469 vs 0.333), macro-AUROC (0.620 vs 0.555), and macro-AUPRC (0.347 vs 0.294) than LLaMA-3-LoRA, whereas LLaMA-3-LoRA retained higher micro-AUROC (0.919 vs 0.889) and micro-AUPRC (0.803 vs 0.735; Table S15 in Multimedia Appendix 1). These findings suggest that part of LLaMA-3-LoRA’s advantage in the full-input setting may reflect its ability to leverage longer contextual information rather than inherent architectural superiority. This observation is consistent with prior work demonstrating that context-aware language models remain relatively robust compared with alternative algorithms when input length is constrained [13]. Additionally, the presence of mixed English and Traditional Chinese text in the clinical narratives may have posed challenges for BERT-based models due to tokenizer and vocabulary limitations, as these models were primarily pretrained on English corpora.

The marked performance gap between the untuned LLaMA-3 and LLaMA-3-LoRA suggests that instruction-guided LoRA fine-tuning effectively restores task-specific alignment that is not preserved during general purpose LLaMA-3 pretraining [19,23]. Qualitative inspection of representative model outputs suggests that LoRA fine-tuning yields may facilitate more concise and clinically grounded rationales (Table S16 in Multimedia Appendix 1; using the same 10 cases as Table S10 in Multimedia Appendix 1). To complement illustrative examples, we performed a blinded qualitative evaluation using independent anesthesiologist reviewers. The LLaMA-3-LoRA demonstrated high factual accuracy (86.25%) and clinical coherence (91.25%), with fair to moderate interrater agreement (Tables S12 and S13 in Multimedia Appendix 1). These findings provide preliminary quantitative support for the reliability of generated rationales, although hallucinations were not completely eliminated and explanations should still be interpreted cautiously. In addition, the untuned model produced instruction-like or template text rather than clinically grounded explanations in 10 out of 40 cases, reflecting limited instruction-following capability and representing a potential source of bias in the qualitative evaluation (Table S12 in Multimedia Appendix 1).

### Data Representation and Input Engineering

It should be noted that structured laboratory and vital sign variables were converted into clinically interpretable text descriptors prior to model input (Table S2 in Multimedia Appendix 1). This preprocessing step externalizes part of the clinical interpretation and may simplify the classification task. In addition, ML baselines were trained using the term frequency inverse document frequency representations rather than native structured tabular variables. Therefore, baseline performance comparisons should be interpreted with these design considerations in mind.

Although narrative reconstruction facilitates integration of heterogeneous clinical inputs and mirrors clinician reasoning, this approach introduces an additional preprocessing requirement that may limit immediate scalability across institutions. In practice, many of the transformation rules applied in this study are derived from standard clinical reference thresholds and could potentially be automated within EHR decision support pipelines. Future work should evaluate whether similar performance can be achieved using hybrid approaches that allow language models to directly interpret raw structured data or incorporate automated feature extraction methods.

### Sensitivity Analysis Excluding Anesthesia Planning Variables

From a causal perspective, anesthesia planning may represent a downstream clinical decision partially influenced by perceived perioperative risk and ASA classification, raising the possibility of proxy-label learning. In our sensitivity analysis, excluding anesthesia planning resulted in a reduction in performance, with LLaMA-3-LoRA achieving an *F*_1_-score of 0.734 (95% CI 0.721-0.746) and MCC of 0.422 (95% CI 0.408-0.436), compared with 0.780 (95% CI 0.769-0.792) and 0.533 (95% CI 0.518-0.546) in the full-input model (Table S17 in Multimedia Appendix 1). Clinically, anesthesia planning reflects integrated evaluation of comorbidities, physiologic reserve, and surgical risk rather than ASA classification alone. These results indicate that anesthesia planning contributes to incremental predictive information but does not fully account for model performance.

### Comparing With Prior Studies

Compared with the prompt-based approach of Chung et al [37], which evaluated instruction-tuned GPT-4 Turbo without additional parameter training, our LLaMA-3-LoRA achieved substantially higher scores across evaluated metrics. Chung et al [37] reported a microaveraged *F*_1_-score of 0.50 (95% CI 0.46-0.53) and an MCC of 0.36 (95% CI 0.32-0.40), whereas LLaMA-3-LoRA attained a microaveraged *F*_1_-score of 0.780 (95% CI 0.769-0.792) and an MCC of 0.533 (95% CI 0.518-0.546). In contrast, the untuned LLaMA-3 model performed worse than the reported GPT-4 results by Chung et al, with a microaveraged *F*_1_-score of 0.073 (95% CI 0.066-0.081) and an MCC of 0.002 (95% CI 0.001-0.002), highlighting the importance of task-specific model adaptation rather than inherent architectural superiority.

Beyond superior predictive accuracy, our pipeline is fully transparent and reproducible: it relies on publicly available LLaMA-3 weights and open-source LoRA fine-tuning code, in contrast to GPT-4 Turbo’s closed model weights. On macroaveraged discrimination, Chung et al [13] reported macro-AUROC 0.865 for fastText and 0.843 for BioClinicalBERT, whereas in our study macro-AUROC was 0.608 for LLaMA-3-LoRA and 0.637 for fastText (Table S5 in Multimedia Appendix 1). Relative to Yoon et al [15], our micro-AUROC for LLaMA-3-LoRA (0.863, 95% CI 0.853-0.872) and ClinicalBERT-ft (0.848, 95% CI 0.838-0.858) were lower than their ClinicalBigBird (0.914, 95% CI 0.909-0.919). This difference likely reflects cohort and label-schema differences (our 5-class, more imbalanced setting vs their 4-class dataset), as well as model and pretraining differences.

In our cohort, traditional classifiers were strong comparators: XGBoost micro-AUROC 0.884 (95% CI 0.875-0.893), SVM 0.880 (95% CI 0.871-0.889), and Random Forest 0.871 (95% CI 0.862-0.880; Table 2). These results are consistent with prior ASA-PS work using structured inputs, including a C-statistic of 0.77 (95% CI 0.766-0.773) for ASA-PS prediction [8] and AUROC 0.884 for an automated perioperative acuity system [14]. Collectively, this indicates that our ML baselines are competitive with the literature and provide a credible benchmark for evaluating LLaMA-3-LoRA.

### Interpretability

Interpretability is another key differentiator. The LoRA-adapted model produces concise, clinically coherent rationales when prompted for explanation. Rationale generation and classification evaluation were conducted using distinct inference configurations; the representative cases shown in Table S10 in Multimedia Appendix 1 were generated under the explanation-augmented prompt and are provided for qualitative illustration only. In these examples (Table S10 in Multimedia Appendix 1), the explanations show that these explanations reference patient age, salient comorbidities, recent cardiac events, and functional limitations—especially in ASA-PS class III and above—to justify the assigned grade. Attention visualizations further confirm that the model focuses on terms such as diabetes, cirrhosis, and stroke, mirroring clinical reasoning (Figures 6 and 7).

Notably, LoRA fine-tuning in this study optimized classification labels only, and free-text rationales were not included as supervised training targets; therefore, explanations were generated during inference using the instruction-following capability of the pretrained base model. However, prior work has shown that explanation-augmented prompting may influence model decision behavior rather than merely reveal it, and that generated rationales can be plausible yet not fully faithful to the underlying prediction process [38]. Accordingly, in this study, explanation-augmented prompts were used exclusively for qualitative illustration and not for quantitative performance evaluation. Despite this limitation, this capacity for transparent, case-specific explanation may facilitate clinician trust and support adoption in real-world perioperative settings.

Attention visualization frequently highlighted clinically relevant comorbidity and physiologic descriptors; however, some high-attention tokens were nonspecific or administrative, suggesting that attention signals may reflect statistical correlations rather than faithful clinical reasoning (Figure 6 and 7; Multimedia Appendix 4). Therefore, attention-based analyses should be interpreted as exploratory qualitative insights rather than definitive mechanistic explanations [34].

### Generative Oversampling for Class Imbalance

To address class imbalance, we evaluated a generative data augmentation strategy targeting a uniform distribution of 3000 cases per ASA class. We generated synthetic samples for minority classes (ASA I, IV, and V) using an equal mix of ChatGPT and Gemini, while downsampling majority classes (ASA II and III). However, this approach resulted in inferior overall performance compared with the baseline LLaMA-3-LoRA configuration, yielding an *F*_1_-score of 0.696 (95% CI 0.683-0.709) and an MCC of 0.408 (95% CI 0.394-0.422; Table S17 in Multimedia Appendix 1). Although this strategy successfully balanced the dataset, the synthetic clinical narratives likely introduced linguistic artifacts or semantic drift. This finding aligns with prior research suggesting that naive generative oversampling can introduce label noise and compromise model calibration without enhancing the representation of rare classes [39,40].

We additionally evaluated a loss-based class reweighting strategy during model training to address class imbalance, with class weights assigned inversely proportional to class frequency. However, this approach did not improve overall performance compared with the baseline LoRA configuration, resulting in a slightly lower micro–*F*_1_-score (0.769 vs 0.780) and MCC (0.506 vs 0.533; Table S17 in Multimedia Appendix 1). Micro-AUROC (0.856 vs 0.863) and micro-AUPRC (0.638 vs 0.653) were also modestly reduced. These findings further support the notion that performance limitations in minority ASA classes may reflect intrinsic ambiguity in ASA severity boundaries rather than sample frequency alone.

### Subgroup Analysis

To assess the consistency of model performance across demographic and clinical strata, we conducted subgroup analyses by sex, age, and comorbidity burden. The LLaMA-3-LoRA model demonstrated stable performance across these subgroups, with *F*_1_-scores of 0.770 for males and 0.793 for females. Across age categories, *F*_1_-scores ranged from 0.711 to 0.848 for age subgroups and across comorbidity-count strata from 0.771 to 0.797. With the exception of the ≥65-year age group, no statistically significant differences were observed, suggesting that the model exhibits generally robust performance across diverse demographic and clinical characteristics (Table S18 in Multimedia Appendix 1).

### Ablation Analysis in LLaMA-3-LoRA

Ablation analysis revealed that sampling parameters, especially temperature and *top_p*, had the greatest effect on model performance. Reducing temperature from 0.9 to 0.1 improved MCC from 0.195 to 0.526 (Table S8 in Multimedia Appendix 1), while lowering *top_p* from 0.9 to 0.1 increased MCC from 0.179 to 0.538 (Table S9 in Multimedia Appendix 1). These results suggest that limiting generation randomness improves the consistency and accuracy of instruction-following behavior. While learning rate (Table S7 in Multimedia Appendix 1, optimal at 3×10⁻⁵) and dropout (Table S6 in Multimedia Appendix 1, optimal at 0.4) also contributed to performance, their relative impact was smaller.

### Limitations

This investigation has several methodological constraints. First, the dataset was markedly imbalanced—ASA-PS classes I, IV, and V accounted for a small fraction of cases—raising the risk of class-specific misclassification and limiting the validity of predictions for these groups. Notably, the model demonstrated markedly reduced sensitivity for ASA class IV, with most cases misclassified as class III (Figure 4). In addition, all ASA class I cases were misclassified in the test set, indicating limited discrimination at the extremes. Consequently, predictions were concentrated within intermediate classes (ASA II-III). This distinction is clinically important because ASA class IV represents patients with life-threatening systemic disease and substantially higher perioperative risk. The reduced sensitivity likely reflects both severe class imbalance and limited preoperative documentation regarding whether systemic disease constitutes a constant threat to life. These findings are consistent with prior ASA classification studies reporting reduced performance for rare severity classes [13,15]. Therefore, the model should be considered an adjunctive decision support or documentation assistance tool rather than a stand-alone risk classification system, and clinician oversight remains essential.

Second, model development and internal validation were confined to a single tertiary medical center, so performance may not translate to institutions with different patient populations, documentation styles, or EHR systems. In particular, our inputs included free text from standardized sections (chief complaint, present illness, admission diagnosis, impression, and treatment plan), whereas anesthesia-related data were derived from structured fields (Table S2 in Multimedia Appendix 1). This reliance on templated narratives may aid portability to institutions with similar section-based documentation, but transfer to settings with free-form or less standardized clinical notes could be more challenging. Approaches such as section header normalization, abbreviation mapping, or light site-specific adaptation may help mitigate these differences.

Third, the study’s retrospective design relies on the accuracy and completeness of clinician-authored notes; inaccuracies in free text or structured fields could introduce bias that the LLM might amplify. In addition, because ASA classification is inherently subjective and demonstrates interrater variability, some discrepancies between model predictions and reference labels may reflect uncertainty in the ground truth rather than true model error [36]. In settings where key physiologic severity descriptors are incompletely documented, future approaches incorporating external medical knowledge sources may help improve reasoning robustness [41]. Additionally, missing laboratory values were treated as normal during preprocessing, which may introduce information bias and limit generalizability, particularly among low-risk patients who did not undergo comprehensive testing. Furthermore, anesthesiologists may incorporate information from external or nondigitized sources (eg, scanned referral documents, outside hospitalization records, or bedside clinical assessment) that were not available in the structured or extractable electronic documentation used for model input. This information accessibility gap may contribute to differences between model predictions and real-world clinical ASA assignment and may limit reproducibility across institutions with heterogeneous documentation practices.

Fourth, although attention weights and exemplars were provided, LLM rationales remain probabilistic and may occasionally generate spurious associations (“hallucinations”), which were not systematically quantified.

Fifth, although no systematic performance differences were observed across sex, age, and comorbidity strata (Table S18 in Multimedia Appendix 1), computational latency and clinician usability, which are essential for real-time perioperative decision support, were not evaluated in this study. A feasible integration pathway would involve deploying the model as a read-only decision-support module in the preoperative clinic, presenting a predicted ASA-PS class with a rationale, allowing one-click clinician confirmation, and capturing feedback for audit and iterative refinement. To facilitate independent validation and external testing, we also deployed a publicly accessible web application using a Gradio-based interface that allows users to input clinical narratives and obtain ASA-PS classes and rationales [42].

Finally, although generated rationales appeared clinically plausible, we did not quantify their alignment with expert judgment. Future work will include prospective multicenter validation, class balancing, demographic fairness audits, real-time deployment in perioperative workflows, and blinded clinician–model rationale alignment mapped to ASA-PS criteria with interrater statistics.

### Conclusions

The LLaMA-3-LoRA model provides a promising alternative to domain-specific and traditional models for ASA-PS classification. It performs well across evaluation metrics, supports explainability through attention and generated rationales, and is computationally efficient for deployment (averaged 2.25 seconds per case). This framework may serve as a basis for scalable, real-world applications in perioperative risk assessment and could be extended to other clinical classification tasks.

## Data Availability

The datasets generated or analyzed during this study are not publicly available due to institutional review board restrictions that prohibit open sharing of patient-level data to protect participant privacy but are available from the corresponding author upon reasonable request.

## Appendix

Multimedia Appendix 1 Supplementary tables including American Society of Anesthesiologists Physical Status (ASA-PS) definitions, feature preprocessing rules, cohort characteristics, detailed model performance metrics, hyperparameter ablation results, and qualitative evaluation of model-generated rationales.

Multimedia Appendix 2 Input content, formatting, and length handling for each model family. All models received the same concatenated instruction + input; only formatting and length limits differed by architecture.

Multimedia Appendix 3 Pairwise McNemar test *P* values comparing classifier error rates across models. Two-sided *P* values were calculated using McNemar test with continuity correction based on paired predictions from the same test set. Predictions were converted into binary outcomes (correct vs incorrect relative to the reference American Society of Anesthesiologists Physical Status label).

Multimedia Appendix 4 Attention-based visualization across representative American Society of Anesthesiologists Physical Status (ASA-PS) classes II and IV. This appendix presents additional attention-based interpretability analyses for representative cases from ASA-PS classes II and IV. These figures complement the main manuscript attention analyses for ASA-PS class III (Figures 6 and 7). Attention visualizations are presented as exploratory qualitative tools to illustrate model focus patterns and do not represent causal decision mechanisms.

Multimedia Appendix 5 Confusion matrices for all evaluated models in American Society of Anesthesiologists Physical Status (ASA-PS) classification. All evaluated classification models, including LLaMA-3-LoRA, untuned LLaMA-3, BioBERT-ft, ClinicalBERT-ft, fastText, Random Forest, Support Vector Machine (SVM), and XGBoost, were included. Each confusion matrix displays model-predicted ASA-PS classes (columns) versus reference clinician-assigned ASA-PS classes (rows), with cell values representing case counts. All confusion matrices were generated using predictions from the fixed held-out test cohort.

## Abbreviations

AC1: agreement coefficient
ASA-PS: American Society of Anesthesiologists Physical Status
AUPRC: area under the precision-recall curve
AUROC: area under the receiver operating characteristic curve
BERT: Bidirectional Encoder Representations from Transformers
EHRs: electronic health record
LLaMA: large language model meta-AI
LLM: large language model
LoRA: low-rank adaptation
MCC: Matthews correlation coefficient
ML: machine learning
NLP: natural language processing
SVM: support vector machine
XGBoost: Extreme Gradient Boosting
