# Supplementary material for: Classifying American Society of Anesthesiologists Physical Status With a Low-Rank–Adapted Large Language Model: Development and Validation Study
Source: J Med Internet Res. 2026 Apr 21;28:e89540. doi: 10.2196/89540 (PMC13146231; doi:10.2196/89540)
Supplement: Multimedia Appendix 2 [file jmir_v28i1e89540_app2.docx]

**Multimedia Appendix 2. Input content, formatting, and length handling for each model family.** All models received the same concatenated instruction + input; only formatting and length limits differed by architecture.

| **Model family** | **Input content** | **Formatting / vectorization** | **Length handling** |
| --- | --- | --- | --- |
| XGBoost / SVM / Random Forest | Instruction + Input | TF-IDF features on the raw concatenated text | Full text (no truncation) |
| fastText | Instruction + Input | Raw concatenated text (word/ngram embeddings) | Full text (no truncation) |
| BioBERT / ClinicalBERT | Instruction + Input | Standard BERT tokenization | 512 tokens (BERT maximum) |
| LLaMA-3-Instruct / LoRA | Instruction + Input | Alpaca-style prompt embedding the same content | Full text within model context window |

Note: “Instruction + Input” refers to the exact same textual content across models; only formatting differs. “Full text” indicates that we did not truncate beyond each model’s inherent context window (BERT limited to 512 tokens; other models used the entire concatenated text available). Figure 2 illustrates the LLaMA prompt.
