# Supplementary figures and images for: Classifying American Society of Anesthesiologists Physical Status With a Low-Rank–Adapted Large Language Model: Development and Validation Study
Source: J Med Internet Res. 2026 Apr 21;28:e89540. doi: 10.2196/89540 (PMC13146231; doi:10.2196/89540)

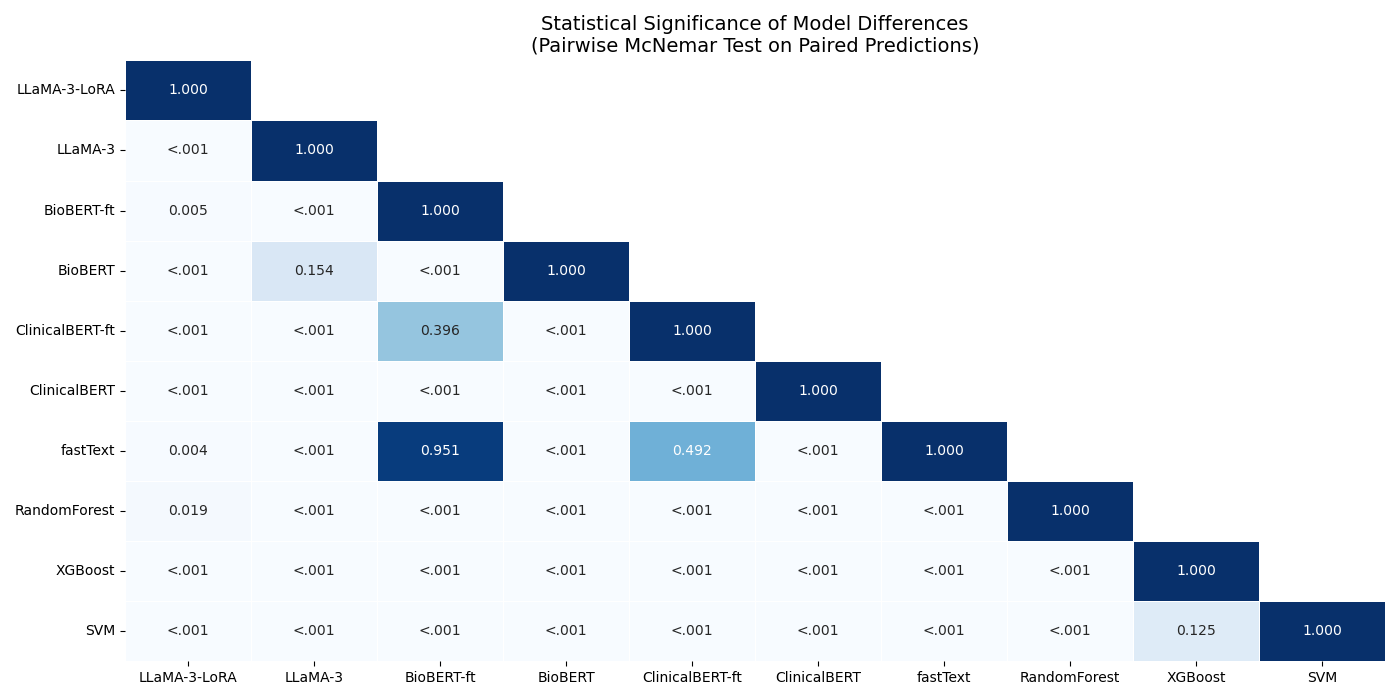

Supplement: Multimedia Appendix 3 [file jmir_v28i1e89540_app3.png]
