# Supplementary material for: Classifying American Society of Anesthesiologists Physical Status With a Low-Rank–Adapted Large Language Model: Development and Validation Study
Source: J Med Internet Res. 2026 Apr 21;28:e89540. doi: 10.2196/89540 (PMC13146231; doi:10.2196/89540)

Figure S1 Top 30 most attended phrases contributing to the ASA-PS output token for one case (ASA-PS Class II) from the test cohort. The lower triangular matrix shows average unidirectional attention weights from each phrase to preceding phrases derived from the final transformer layer. Brighter regions indicate stronger attention. Medical terms demonstrate localized clustering, suggesting contextual association between severity-related descriptors.

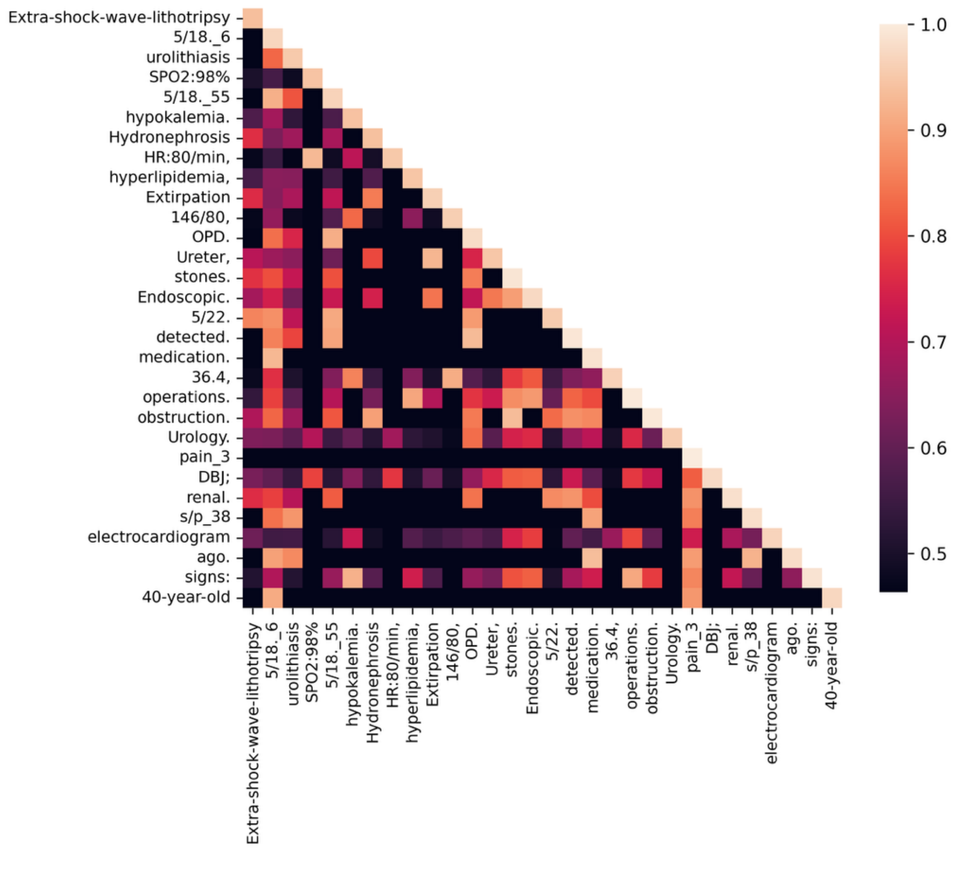

Figure S2. Attention heatmap between top 30-word phrases in the input text for one case (ASA-PS Class II) from the test cohort. Bar height represents cumulative attention directed toward each phrase by the final output token. Highlighted phrases primarily reflect comorbidities, physiologic abnormalities, and demographic features relevant to perioperative risk classification.

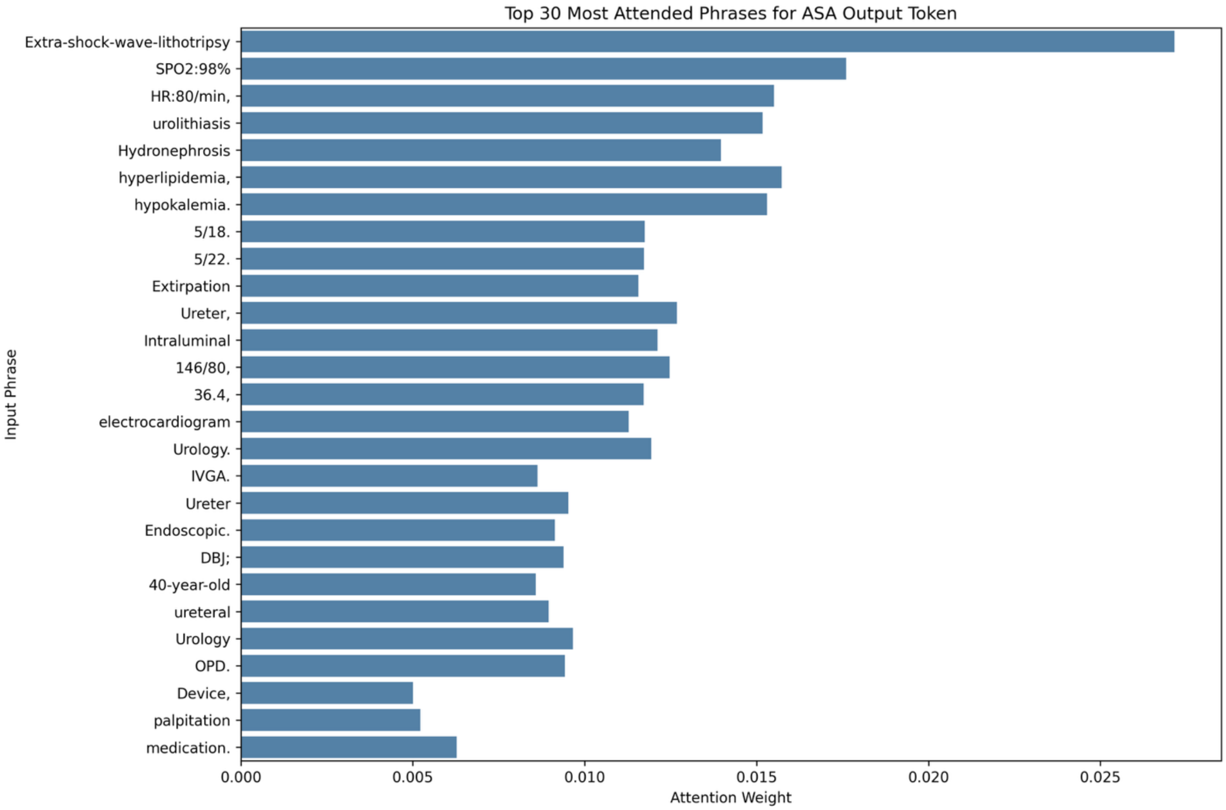

Figure S3 Top 30 most attended phrases contributing to the ASA-PS output token for one case (ASA-PS Class IV) from the test cohort. The lower triangular matrix shows average unidirectional attention weights from each phrase to preceding phrases derived from the final transformer layer. Brighter regions indicate stronger attention. Medical terms demonstrate localized clustering, suggesting contextual association between severity-related descriptors.

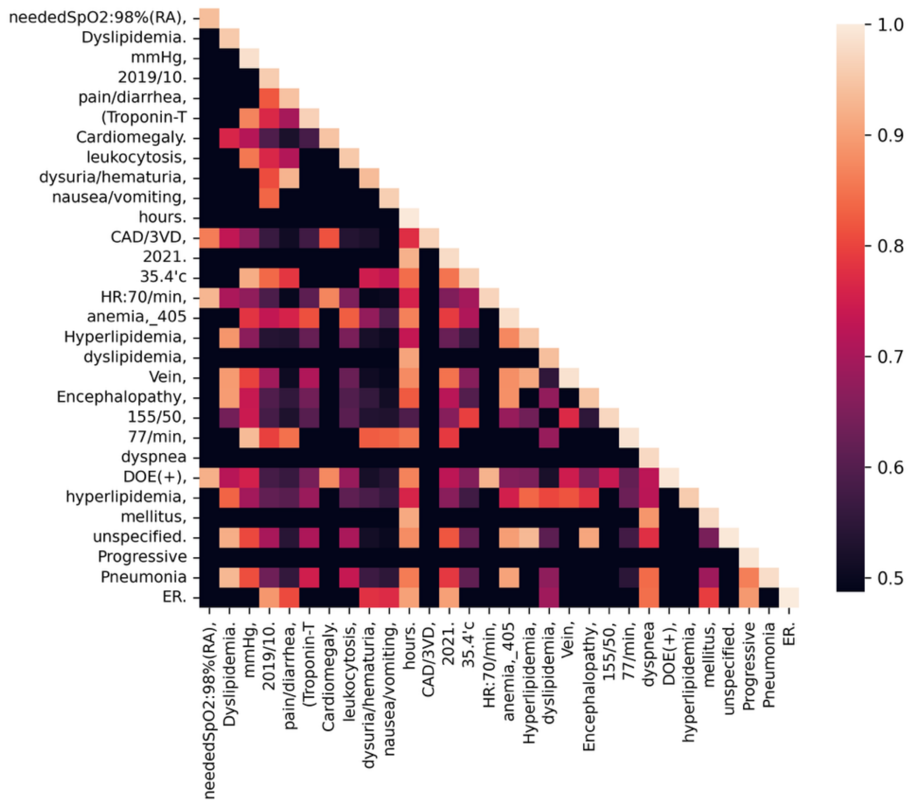

Figure S4. Attention heatmap between top 30-word phrases in the input text for one case (ASA-PS Class IV) from the test cohort. Bar height represents cumulative attention directed toward each phrase by the final output token. The highlighted phrases reflect model attention to severe comorbidity descriptors, physiologic instability indicators, and functional impairment features associated with higher perioperative risk.

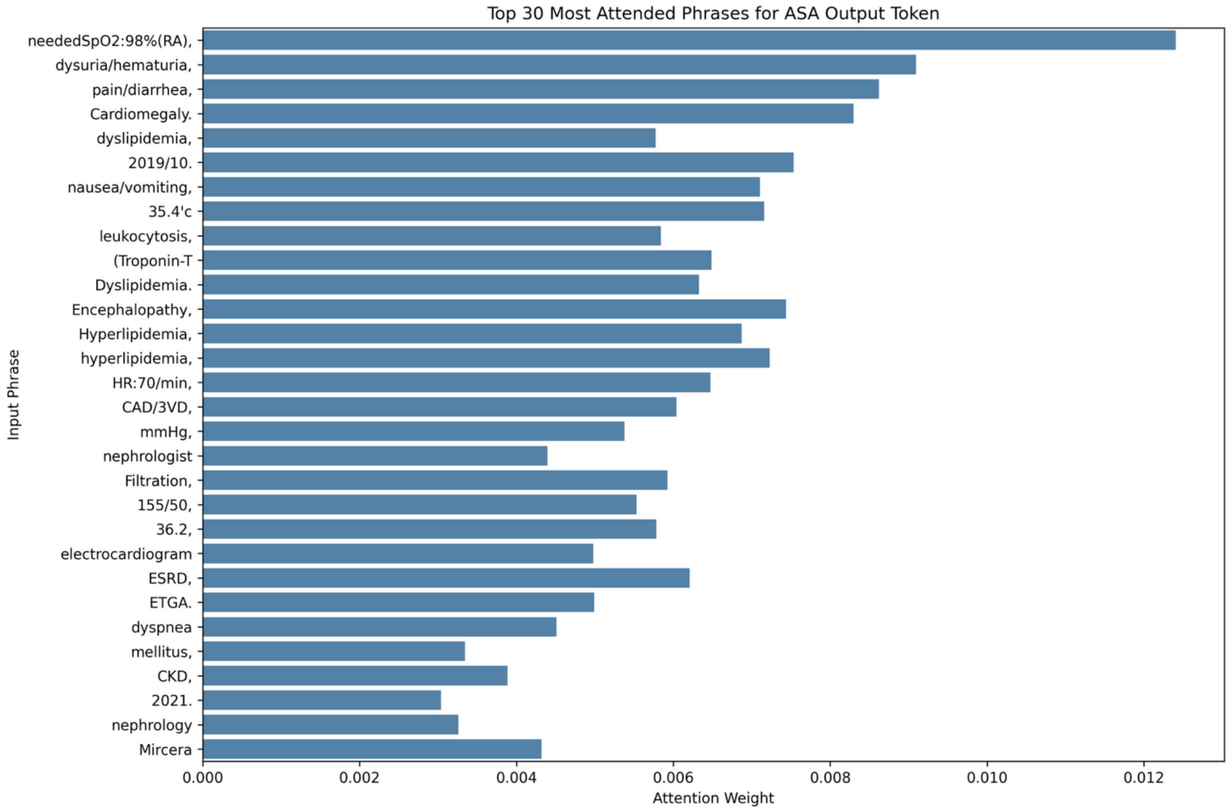

Supplement: Multimedia Appendix 4 [file jmir_v28i1e89540_app4.pdf]
