# Supplementary material for: Classifying American Society of Anesthesiologists Physical Status With a Low-Rank–Adapted Large Language Model: Development and Validation Study
Source: J Med Internet Res. 2026 Apr 21;28:e89540. doi: 10.2196/89540 (PMC13146231; doi:10.2196/89540)
Supplement: Multimedia Appendix 5 [file jmir_v28i1e89540_app5.pdf]

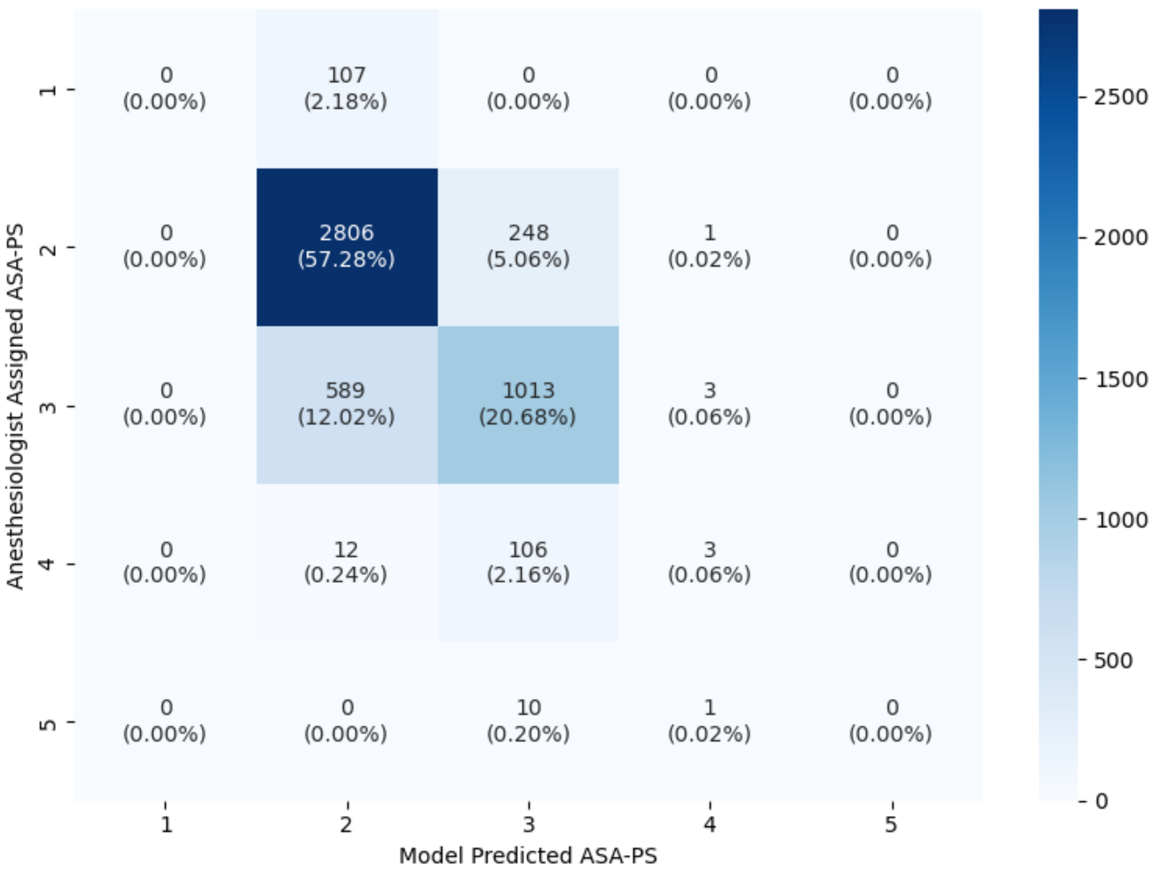

Figure S1. Confusion matrix for ASA-PS classification using the LLaMA-3-LoRA model.

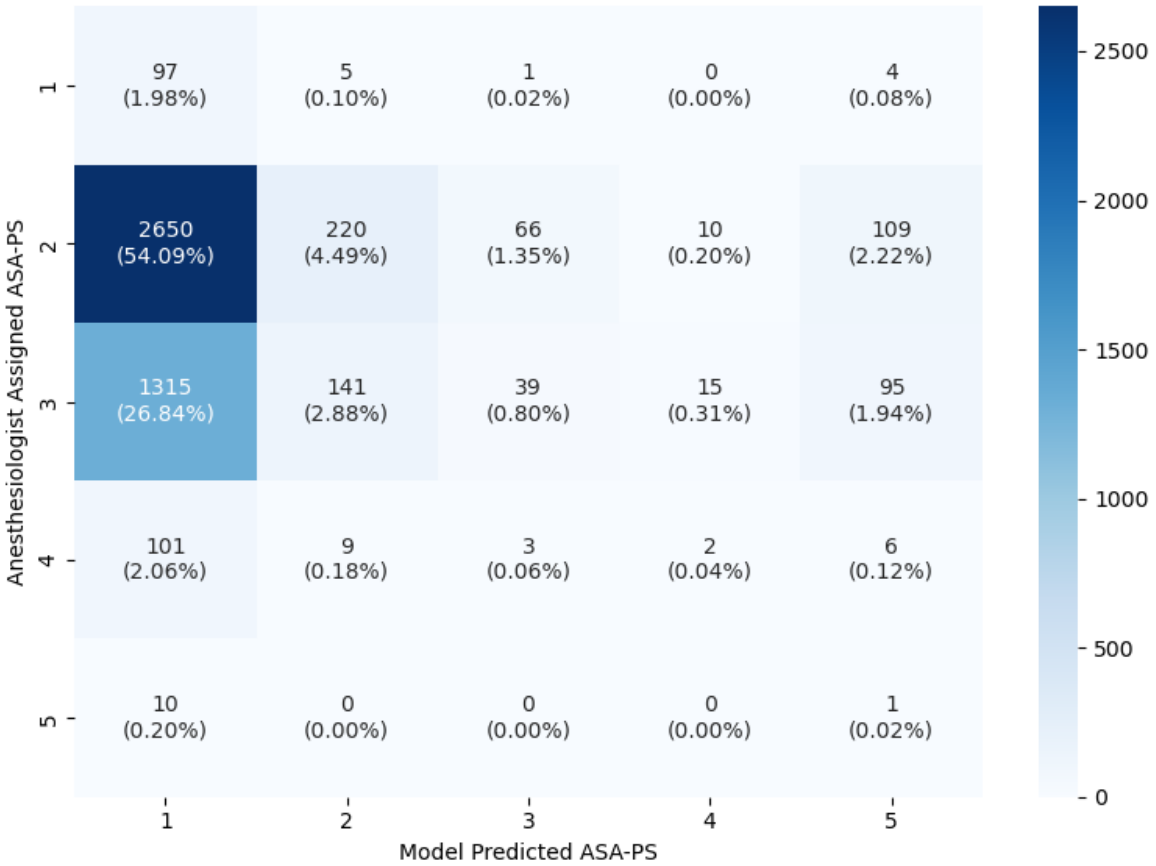

Figure S2. Confusion matrix for ASA-PS classification using the LLaMA-3 model.

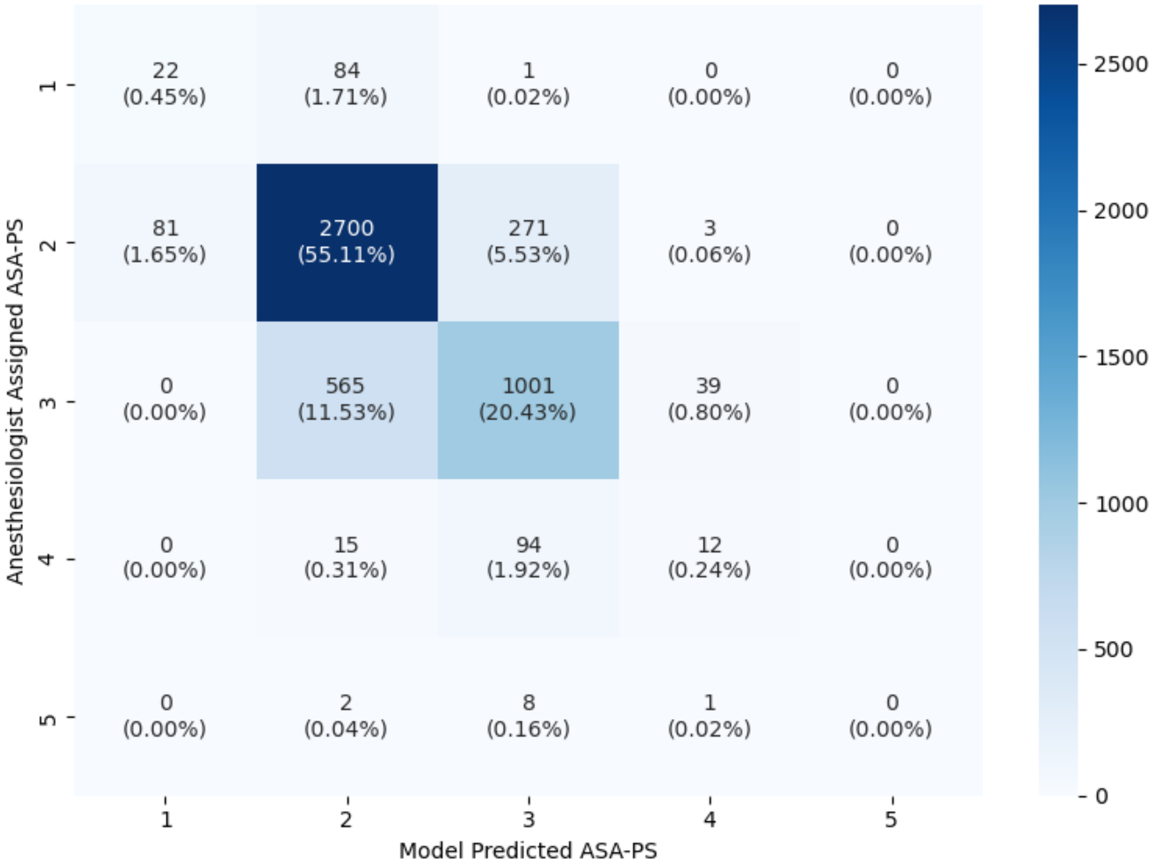

Figure S3. Confusion matrix for ASA-PS classification using the BioBERT-ft model.

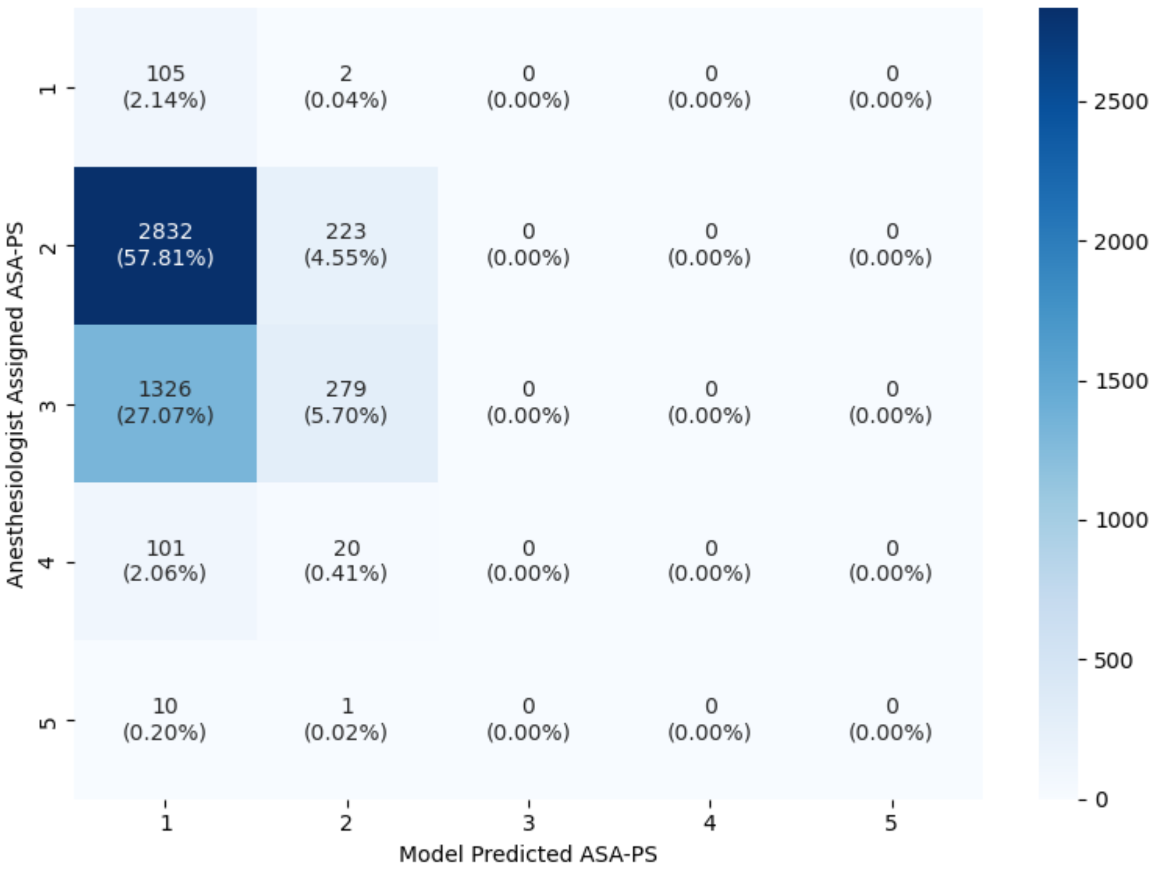

Figure S4. Confusion matrix for ASA-PS classification using the BioBERT model.

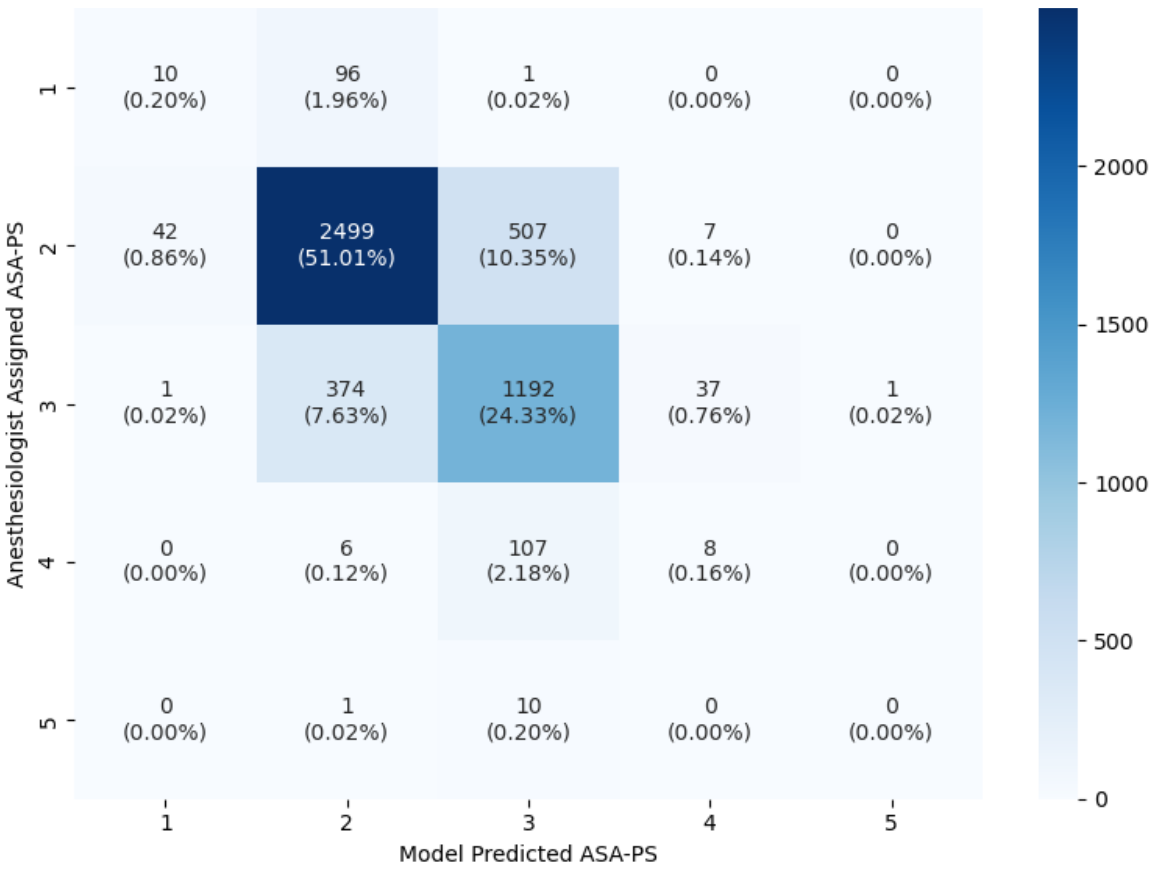

Figure S5. Confusion matrix for ASA-PS classification using the ClinicalBERT-ft model.

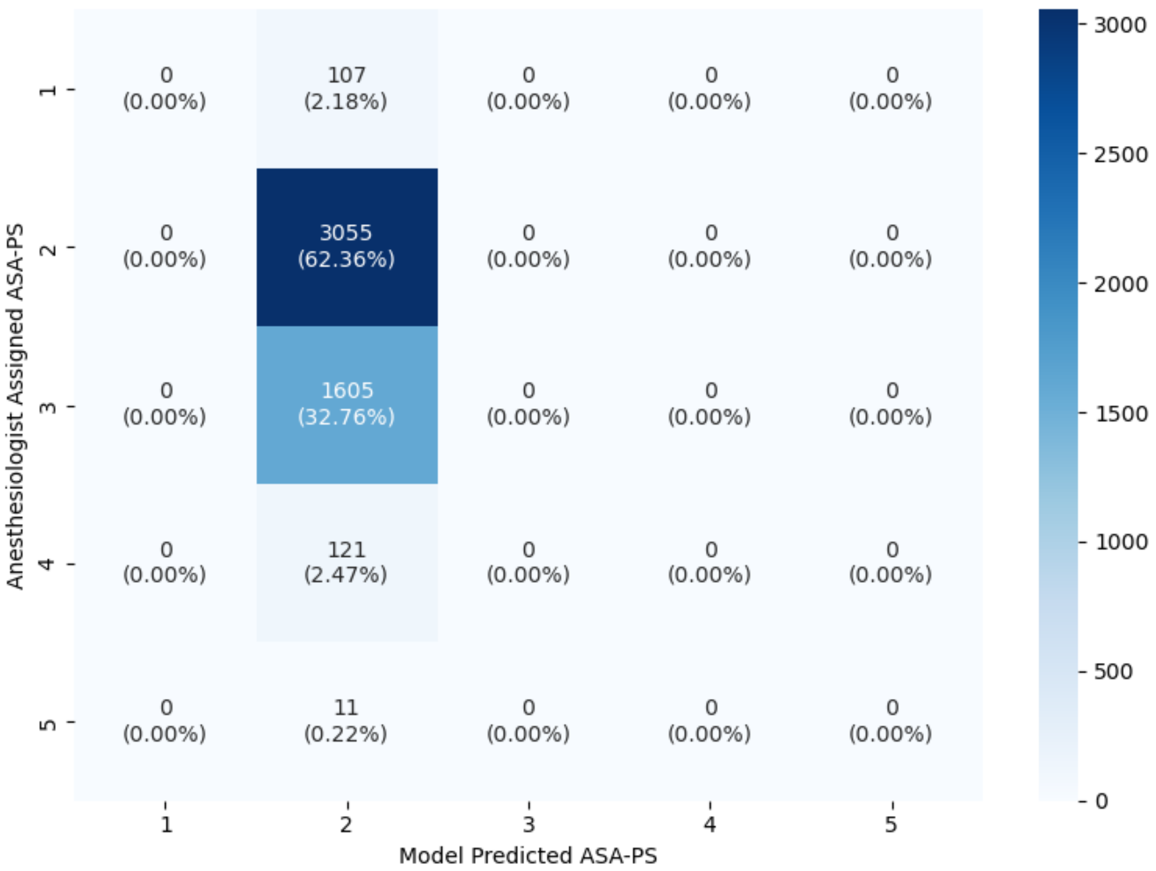

Figure S6. Confusion matrix for ASA-PS classification using the ClinicalBERT model.

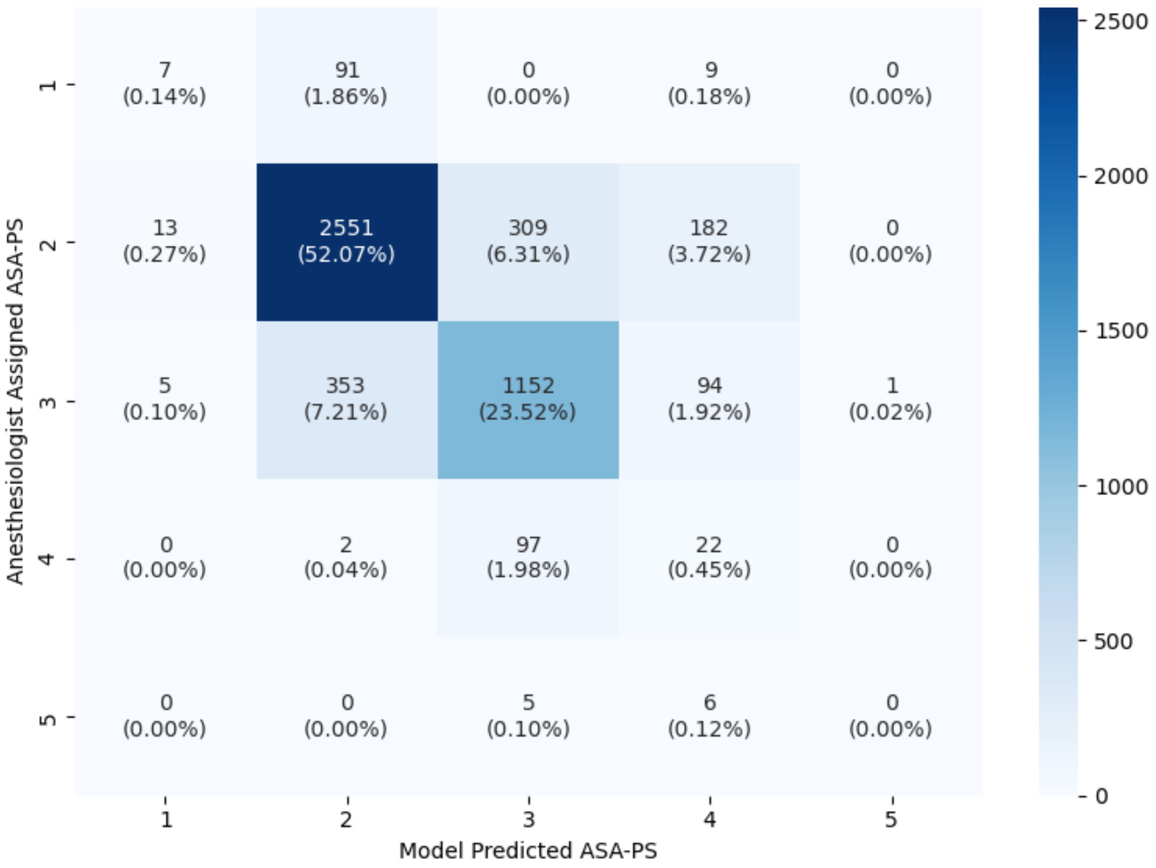

Figure S7. Confusion matrix for ASA-PS classification using the fastText model.

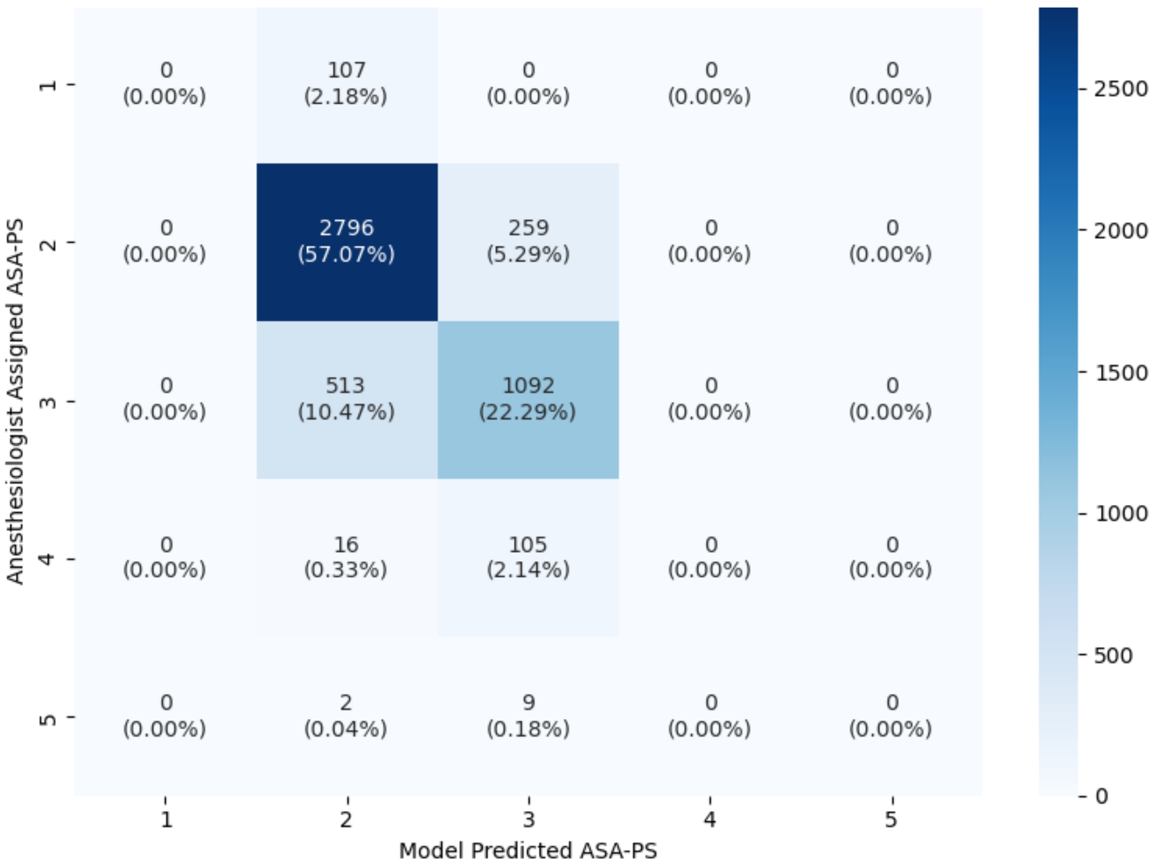

Figure S8. Confusion matrix for ASA-PS classification using the random forest classifier.

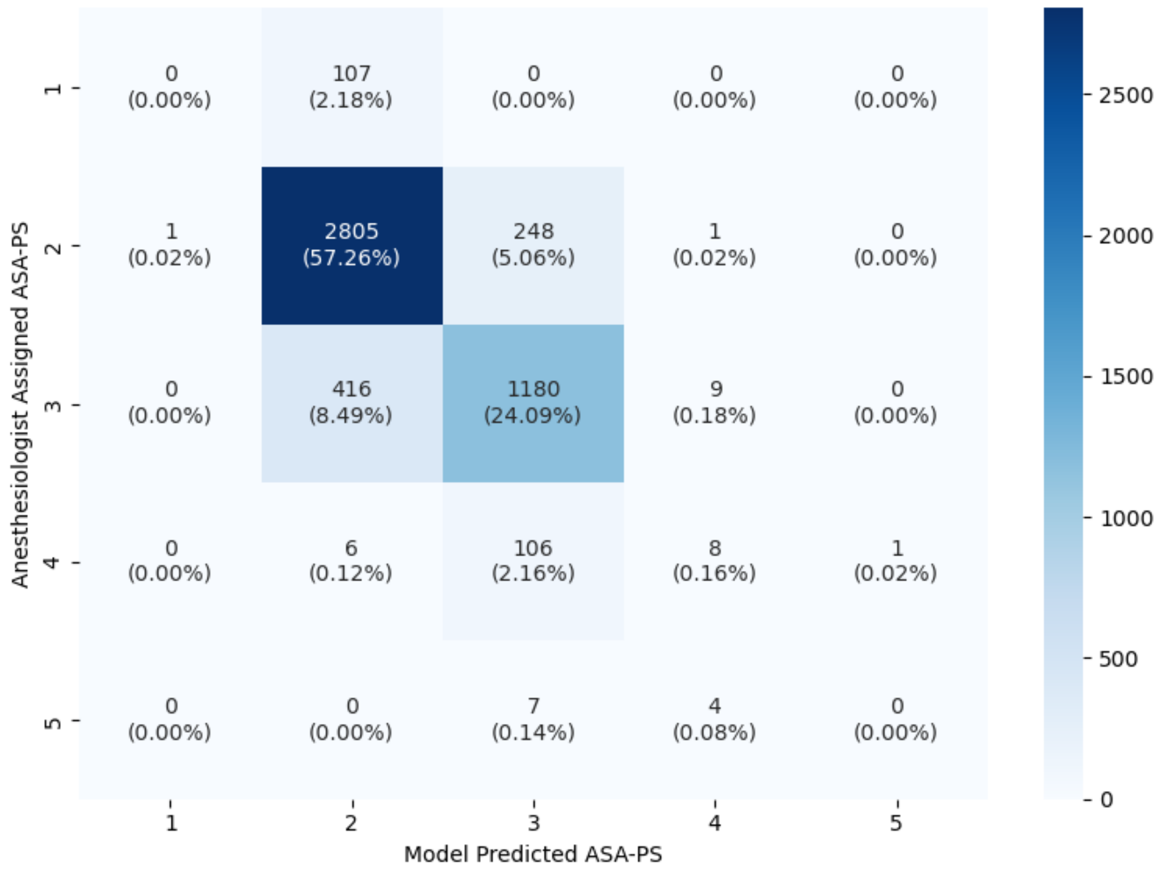

Figure S9. Confusion matrix for ASA-PS classification using the XGBoost model.

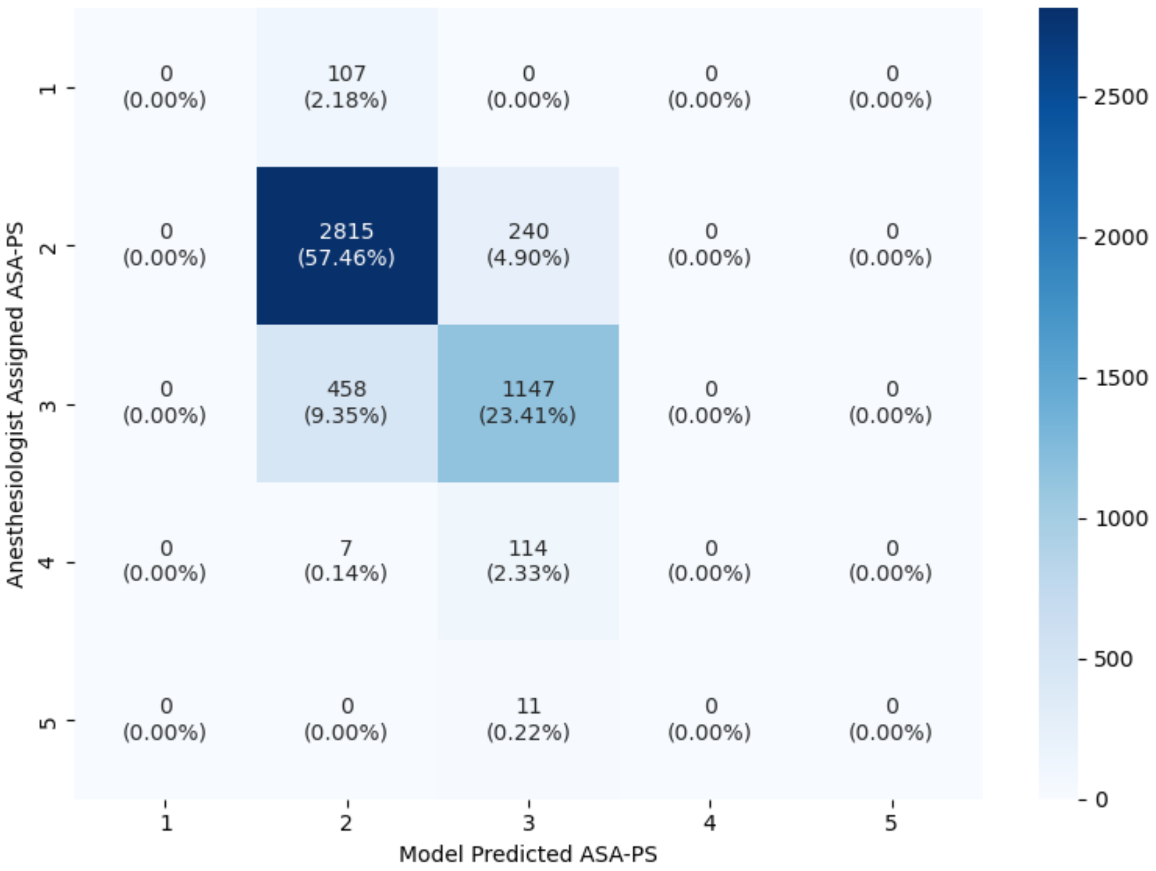

Figure S10. Confusion matrix for ASA-PS classification using the support vector machine.
